# Upper Extremity Return to Sport Functional Testing: A Systematic Review

**DOI:** 10.1186/s40798-026-00984-4

**Published:** 2026-02-23

**Authors:** Marisa Pontillo, Eric Bellm, Patrick Barber, Matthew Gauthier, Casey Unverzagt, George Davies

**Affiliations:** 1 Extremity Trauma and Amputation Center of Excellence, Defense Health Agency, 7700 Arlington Blvd, Falls Church, VA 22042 USA; 2 Department of Physical and Occupational Therapy, Chiropractic Services, and Sports Medicine, Naval Medical Center San Diego, San Diego, CA 92134 USA; 3 Department of Physical Therapy and Athletic Training, Saint Louis University, Saint Louis, MO 63104 USA; 4 Department of Orthopaedics and Physical Performance, University of Rochester Medical Center, Rochester, NY 14618 USA; 5 Department of Physical Therapy, College of Applied Health Sciences, University of Illinois at Chicago, 1640 W Roosevelt Rd, Chicago, IL 60608 USA; 6 Department of Physical Therapy, Robbins College of Health and Human Sciences, Baylor University, 900 Washington Ave., Waco, TX 76701 USA; 7 University of Texas-Rio Grande Valley, Edinburg, TX 78539 USA; 8 Gundersen Health System Sports Medicine, La Crosse, WI 54650 USA

**Keywords:** Upper extremity, Measurement, Functional test, Return to sport, Psychometric properties

## Abstract

**Background:**

Upper extremity return to sport (RTS) assessments are not standardized with respect to which metrics to use, if the metrics are appropriate for all populations and levels of competition, and what constitutes good or poor test performance. Subsequently, clinicians may utilize suboptimal metrics to evaluate RTS readiness, or forgo objective criteria altogether. The purpose of this study was to examine the psychometric properties (reliability, agreement/measurement error, hypothesis testing/construct validity, criterion/predictive validity, responsiveness) of upper extremity functional tests used to assess RTS readiness.

**Methods:**

This systematic review followed PRISMA guidelines, with all studies included assessed via the PEDro scale. Literature searches covering PubMed, Google Scholar and Medline databases were completed through November 2024. Studies focusing on the reliability and/or validity of upper extremity functional tests in athletes were included.

**Results:**

A total of 5166 studies were identified; 60 studies met criteria for data extraction. Among the identified tests, the Single Arm Shot Put test (SASP), Closed Kinetic Chain Upper Extremity Stability Test (CKCUEST), and the Upper Quarter Y-Balance test (UQY) were the most frequently investigated. The SASP emerged as consistently reliable (the preponderance reporting ICC > 0.90), with construct validity evidenced by correlations with upper extremity isokinetic torque and performance on other functional tests. The CKCUEST demonstrated good to excellent reliability across age, sex, and sport, including individuals with shoulder pain (ICC = 0.73–0.98). Construct validity was established via strong correlations with grip and isokinetic upper extremity strength (*P* < 0.01), and concurrent validity when compared to other upper extremity functional tests. The CKCUEST also demonstrates predictive validity for determining future upper extremity injury risk, and discriminant validity distinguishing individuals with and without current shoulder injury. The UQY yielded mixed reliability, with ICC = 0.47–0.97; additionally, numerous studies found no significant relationships between the UQY and other measures (strength and/or other upper extremity functional tests). The athletic shoulder test (ASH) is an emerging test designed to evaluate isometric strength of the upper body in 3 positions with the athlete prone. Across all testing positions, the ASH has excellent test–retest reliability (ICCs ranging from 0.94 to 0.98), and has demonstrated high concurrent validity when a sphygmomanometer or hand-held dynamometer is utilized instead of a force plate.

**Conclusions:**

This is the first extensive systematic review examining the psychometric properties of commonly administered upper extremity functional tests used to determine RTS criteria, with 60 articles analyzed. The CKCUEST and SASP demonstrate consistent reliability and validity across multiple athletic populations. Understanding strengths and limitations of upper extremity functional tests aids clinicians in choosing appropriate assessments for RTS across age, sex, sport, and level, as well as healthy and injured athletes.

**Supplementary Information:**

The online version contains supplementary material available at 10.1186/s40798-026-00984-4.

## Key Points

- This updated systematic review examining the psychometric properties of commonly administered upper extremity functional tests used to determine RTS criteria across 60 articles.
- The Single Arm Shot Put test and Closed Kinetic Chain Upper Extremity Stability Test demonstrate consistent reliability and validity across multiple athletic populations; the Closed Kinetic Chain Upper Extremity Stability Tests additionally is the only test that demonstrates predictive validity.
- Understanding strengths and limitations of upper extremity functional tests aids clinicians in choosing appropriate assessments for RTS across a variety of athletic populations and sports.

**Supplementary Information:**

The online version contains supplementary material available at 10.1186/s40798-026-00984-4.

## Introduction

It is paramount that clinicians utilize objective metrics when determining return to sport (RTS) readiness after injury; however, substantial debate persists regarding which metrics should guide these decisions. Ideally, RTS assessments would have functional components which replicate the specific movements and physiological demands of the athlete’s sport. This is nonetheless complicated by myriad factors, including injury type and location, sport type, level of participation, and other contextual factors must be considered when assessing readiness. This decision is particularly challenging in upper extremity RTS testing, which historically has been underrepresented in the literature. As a result, standardized upper extremity RTS tests and measures are notably lacking, and no universally accepted battery currently exists to clear an athlete to participate in their previous sport(s) after injuries.

Currently, most upper extremity RTS guidelines are typically derived from studies of a specific surgical procedure [1–5] or a particular patient population. A recent data-driven case series proposed a RTS algorithm for football players returning to sports after shoulder injuries [6]. However, most information describing upper extremity RTS is published as clinical commentaries based on expert opinion to guide decision making [7–9]. A recent noteworthy consensus statement [8] addressed six different domains of RTS testing: (1) pain; (2) active range of motion; (3) kinetic chain; (4) psychological readiness; (5) sport specificity; and (6) strength, power, and endurance. This consensus statement recommended nine different physical performance tests to be considered in a RTS battery. A similar and complementary Delphi study [10] revealed that clinicians who treat athletes after upper extremity injuries agree that functional testing is important to help determine RTS readiness, yielding objective measures of power, stability, and function. However, there is much less clarity as to which test(s) are the most pertinent to include. Furthermore, in a recent survey study, clinicians treating athletic patients with upper extremity injuries stated they wanted to use more functional testing; however, over 50% of the 498 respondents highlighted a lack of understanding of the current research as a significant barrier to implement functional testing into clinical practice [11].

A previous systematic review published in 2016 [12] identified the psychometric properties (reliability, agreement/measurement error, hypothesis testing/construct validity, criterion/predictive validity, responsiveness) of six upper extremity functional tests: the Closed Kinetic Chain Upper Extremity Stability Test (CKCUEST), Bilateral Seated Shot put, Unilateral Seated Shot Put, Medicine Ball Throw, Modified Push Up Test, and One-Arm Hop Test. Eleven articles were assessed, though several of the identified functional tests only had a single reference article. The review found that the CKCUEST and the Unilateral Seated Shot Put Test provided moderate positive evidence for their utilization when making RTS decisions. However, at the time of this review, the utility of physical performance tests at large was chiefly unsubstantiated in the literature; therefore, the authors concluded by recommending further investigation.

A second systematic review published in 2024 [13] aimed to provide an update to the 2016 review by assessing psychometric properties (specifically reliability and validity) of upper extremity functional tests published through March 2021. This study analyzed 13 functional tests across 15 articles: Arm-Jump Board Test, CKCUEST, Finger Hang Test, Medicine Ball Explosive Power Test, One-Arm Hop Test, Posterior Shoulder Endurance Test, Pull-Up Shoulder Endurance Test, Repetition to Failure Assessment, Seated Medicine Ball Throw Test, Unilateral single-arm shot-put test (SASP), Unilateral Seated Shot-Put Test, Two-Arm Bent Hang Test, and Upper Limb Rotation Test.

This review found that only the CKCUEST and SASP are sufficiently reliable to be used clinically in the athletic population, and that the Seated Medicine Ball Throw is valid for the assessment of upper extremity power. The authors caution against use of other tests included in their review due to insufficient measurement properties and recommend further investigation into these functional tests. However, due to the authors’ exclusion criteria, specifically the criterion excluding studies that involved “technology-dependent” instrumentation, several prominent upper extremity functional tests were omitted from their review. This criterion introduced a substantial gap, as technologically instrumented functional tests—such as those employing force plates—are increasingly prominent in contemporary performance assessment. The present review aims to address this gap by updating and broadening prior reviews to include both equipment-free and instrumented functional tests, enabling clinicians to select assessments most appropriate for their specific athletic populations.

When considering which functional test(s) are most suitable for inclusion in a RTS algorithm, reliability, validity, and generalizability must be assessed. Most studies exhibited substantial heterogeneity with respect to athletes’ age, sport, and competition level. Many validity studies focus on concurrent validity (i.e., comparison with other metrics); very few demonstrate predictive validity [14]. Unfortunately, variations in testing protocols including disparities in testing instruction, procedure, and scoring, further diminish the generalizability and clinical applicability of many tests.

Without a data-driven framework to assess upper extremity impairments, athletes may experience a potential delay in their return to previous level of sport or an unacceptable risk of reinjury upon RTS. In some cases, suboptimal or entirely absent objective criteria may be used to progress athletes through rehabilitation and determine clearance, a concern supported by evidence that fewer than half of surveyed clinicians employ functional tests when making upper extremity RTS decisions [11]. This could potentially lead to performance decrements, a higher incidence of re-injury, or referral for surgical intervention, secondary to persistent residual impairments.

**Fig. 1:**
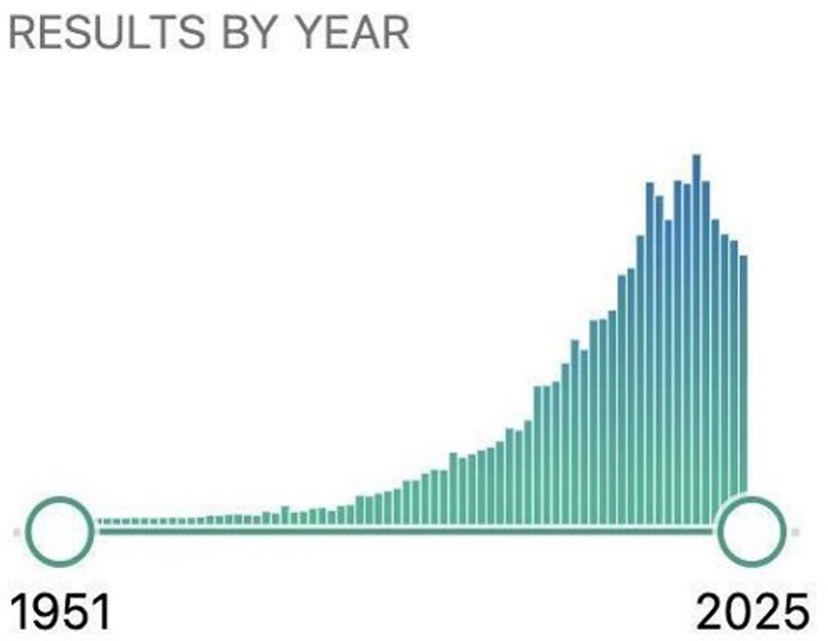
Peer-reviewed publications identified using the search term “upper extremity functional test”. *Source*: PubMed. Retrieved: December 22, 2024

Recent literature [12, 13] has continued to investigate the properties of various upper extremity functional tests; however, these have not been systematically appraised in aggregate. Additionally, novel upper extremity functional tests, including tests requiring specialized clinical or biomechanical instrumentation (e.g., force plates) are continually being developed and evaluated to potentially find solutions in areas for which current tests are lacking. This has led to a rapid rise in general interest and as a result, peer-reviewed publications on this topic (Fig. 1). Collectively, this underscores the need for a robust systematic review of existing upper extremity functional tests to support evidence-based RTS decision-making.

The aim of this systematic review was to examine the psychometric properties of published upper extremity functional performance tests which are used to assess RTS readiness in athletic populations, including reliability, agreement/measurement error, hypothesis testing/construct validity, criterion/predictive validity, and responsiveness.

## Methods

### Eligibility Criteria

This systematic review was conducted in accordance with the Preferred Reporting Items for Systematic Reviews and Meta-Analyses (PRISMA) Statement [15]. Studies were considered if they examined the measurement and/or psychometric properties (i.e., reliability, validity) of at least one upper extremity functional test. As some tests are bilateral and limb symmetry cannot be calculated, studies including normative/reference values and/or sex- and sport-based differences were also included. Inclusion and exclusion criteria were set via the participants, intervention, comparisons, outcomes and study design (PICOS) approach [16]. The inclusion criteria were: (1) original article published in a peer-reviewed journal; (2) the study involved subjects who were at least 12 years of age; (3) the study investigated the psychometric properties or use of an upper extremity test or battery of upper extremity tests; (4) the upper extremity functional test is typically used in active individuals or athletes to assess return to play status; and (5) full text was available in English. Studies were excluded if: (1) the study did not contain an experimental study design with the original data; including, but not limited to, books, systematic or narrative reviews, case studies, opinion pieces, or were published in non-peer reviewed sources; (2) the study involved subjects who were less than 12 years of age; (3) the study used an upper extremity functional test to determine the efficacy of an intervention; (4) the upper extremity functional test is not typically used in athletic populations or is used for non-orthopedic injuries/pathologies; (5) the test(s) assessed only one impairment (e.g., strength or endurance); and (6) the full text was not available in English.

### Literature Search Strategy

Literature searches were conducted in three electronic databases including PubMed, Google Scholar, and Medline, from inception until November 2024, with no year restriction applied to the search strategy. The following key terms (and synonyms searched for by the MeSH database) were included and combined using the operators “AND”, “OR”: “upper extremity” OR “shoulder” AND “functional test” OR “physical performance test” OR “return to sport”; by individual test: “closed kinetic chain upper extremity stability test”, “CKCUEST”, “shot put” AND “seated” OR “single arm”, “upper quarter Y balance test”, “Y balance test” OR “YBT” AND “upper extremity” OR “upper quarter”, “push up”, “one arm hop”, “medicine ball throw”, “wall throw test”, “ball drop test”, “pulling assessment”, “pulling test”, “pushing assessment”, “pushing test”, “functional throwing index”. In addition, the reference lists and citations (via PubMed or Google Scholar) of the identified studies were explored to potentially detect additional relevant research studies. The search for published studies and study selection was performed independently by the primary author (MP).

### Study Selection

**Fig. 2:**
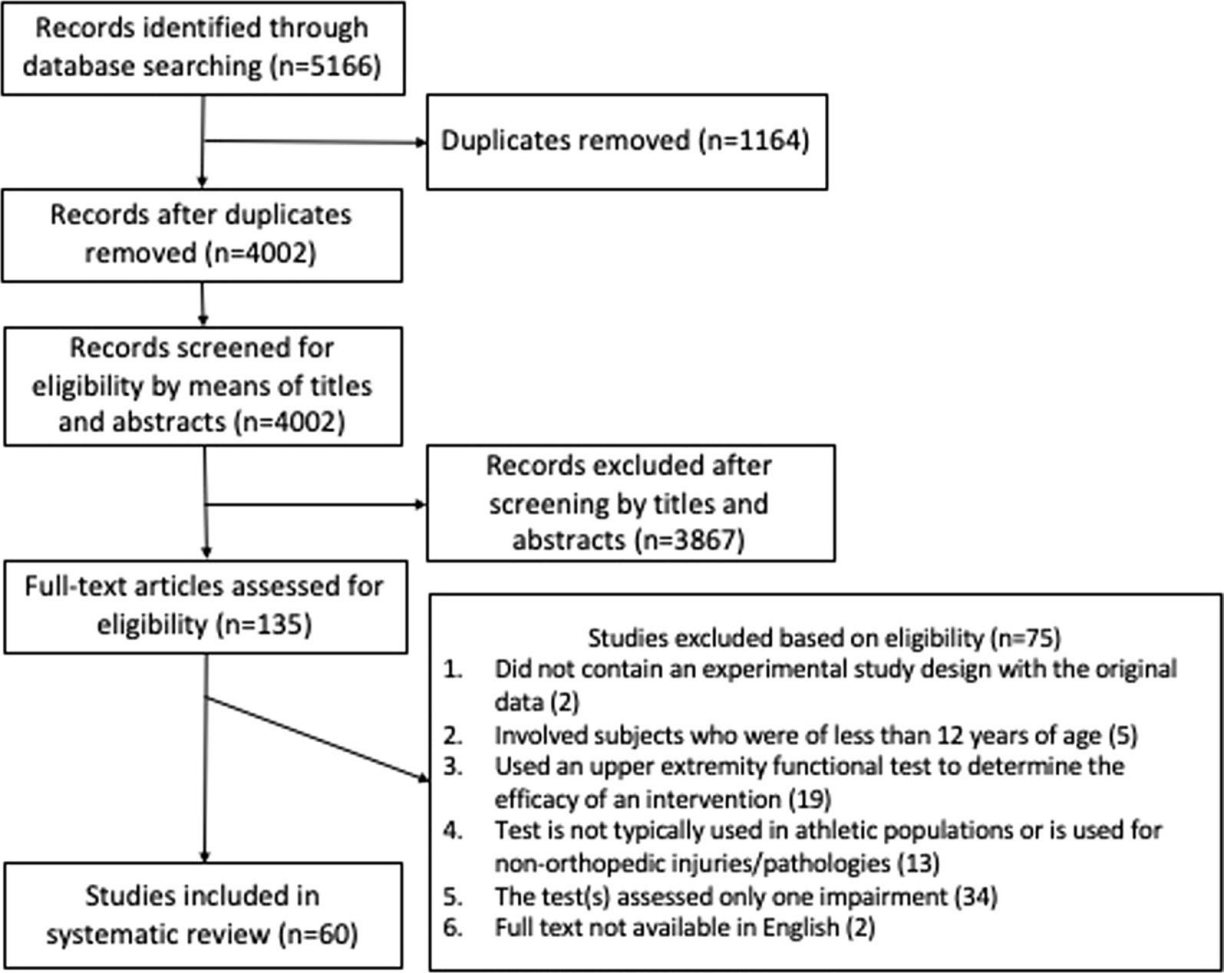
Flow chart for study selection

The screening and study selection were done by the author based on the prior defined inclusion and exclusion criteria and the identified items for assessing the properties or use of an upper extremity test or battery of upper extremity tests. If the title and the abstract of the article showed potential relevance, the full text was examined (Fig. 2). Full text of included articles were downloaded into subfolders, categorized by individual test, and shared with the coauthors. The authors who performed data extraction served as a second line of review to ensure each study screened met inclusion and exclusion criteria prior to extracting the data into Additional file 1: Appendix S1. If necessary, any questions or disagreements regarding study inclusion were resolved by the first author.

### Data Extraction

Once the inclusion/exclusion criteria were applied, data extraction was conducted to collect information about PICOS following PRISMA methodology.

The following relevant data from each study were extracted: study details (author, year of publication, country), study design, study population (sample size, age, participants’ sex, injured or healthy), sport/level (if applicable), which upper extremity functional test(s) were examined, inclusion/exclusion criteria, comparator(s) (if applicable), statistical analysis, outcome measures, results, and clinical application. Three authors performed data extraction and summarizing (chart: Additional file 1: Appendix S1), and provided the COSMIN score. If necessary, disagreements regarding final COSMIN scores were resolved by the first author.

### Quality Assessment

The methodological quality of the included studies was assessed using the COSMIN Risk of Bias checklist. Each article was assessed with respect to the criteria for the property or properties (e.g., reliability, measurement error, etc.) and the final “score” for each article was reported on the 4 point scale (very good, adequate, doubtful, inadequate), with the lowest score being reported. The quality of the articles in the summary (Additional file 1: Appendix S1) was cross-checked by four additional authors (EB, PB, MG, CU), one of whom performed a final quality assessment for the entire article summary (CU). If necessary, disagreements regarding if the study met inclusion and exclusion criteria were resolved by the senior author (GD).

### Equity, Diversity, and Inclusion Statement

All studies which met the inclusion criteria were included, regardless of participant sex/gender, race/ethnicity, and socioeconomic level. The author team included individuals with various levels of experience, individuals from marginalized backgrounds, and perspectives from multiple disciplines.

## Results

From our initial search, 60 studies (Fig. 2) were included in this review (Additional file 1: Appendix S1). These studies originated in Belgium (2), Brazil (4), Canada (1), France (2), Germany (3), India (1), Iran (1), Korea (1), New Zealand (2), Norway (2), Tunisia (1), Turkey (1), United Kingdom (7), and United States (32). The earliest publication date was 1994.

### Single Arm Shot Put

Six articles examined the reliability and five articles examined the validity of the SASP test. The SASP demonstrated an intersession/test–retest reliability (via interclass correlation coefficients [ICCs]) ranging from 0.65 to 0.98 in populations of active adults and athletes [17–22]. Additionally, the SASP demonstrated intra-examiner reliability of ICC_2,3_ = 0.94 and inter-examiner reliability of ICC_2,3_ = 0.97 in a population of recreational athletes with shoulder pain [23]. One study compared performance of the SASP test with the subject seated on the floor to performance with the subject seated in a chair, finding that reliability was higher when performed on the floor (ICC = 0.95–0.97 on the floor, 0.74–0.91 on a chair) [24]. It was also found that female subjects demonstrated significantly greater performance when seated in a chair compared to the floor (*P* < 0.001), possibly related to the presence of systematic error found when performing in a chair (*P* = 0.011 on the dominant arm) [24].

Strong relationships have been observed between SASP and both shoulder flexion and elbow extension isokinetic peak torques (*r* ≥ 0.80, *P* < 0.001) [25] as well as grip strength (*r* = 0.83, *P* < 0.05) [26]. Moderate relationships have been observed between SASP and isometric strength of shoulder flexion, extension, adduction, abduction, internal rotation, and external rotation (*r* ranged from 0.47 to 0.67, *P* < 0.05) [26]. Two articles examined the relationship between SASP and bench press power in female collegiate varsity athletes (*r* = 0.38, *P* < 0.05) [27] and in Division II football players for both absolute (*r* = 0.51) and relative (*r* = 0.66) bench press power [28]. A moderate relationship existed between SASP performance and shoulder horizontal adduction range of motion (*r* = 0.47, *P* = 0.45), but range of motion in all other planes exhibited no correlation [26]. The quality of SASP studies are all “doubtful” or “adequate” (Additional file 1: Appendix S1).

### Closed Kinetic Chain Upper Extremity Stability Test

Ten articles examined the reliability, nine articles examined the validity, and two articles reported the normative scores of the CKCUEST. Populations included athletes, physically active individuals, sedentary individuals, and ages spanning adolescent to adult. Intratester reliability ranged from ICC = 0.86–0.97 and test–retest reliability ranged from 0.73 to 0.93 [17, 20, 21, 29–34]. This test was also found to be reliable in individuals with subacromial impingement syndrome (ICCs ranged from 0.86 to 0.97) [29, 32]. A 2023 reliability study found a significant between-session improvement for males (*P* = 0.003) and females (*P* < 0.001), suggesting a possible learning effect [21]. Minimal detectable change (MDC) values ranged from 2.05 to 4.76 [29–31]. One article found that reliability improved and MDCs were smallest utilizing a setup position where the hand separation is 50% of the individual's height over the standard 36-inch distance [30]; additionally, the relationship between arm length and CKCUEST score (*r* = 0.81; *P* < 0.01) indicates that normalizing score to arm length may improve the test’s accuracy [35]. This relationship is further underscored by an article finding a positive relationship between height and CKCUEST power score (*r* = 0.66 among males and *r* = 0.43 among females; *P* < 0.01) [36]. The CKCUEST’s reliability is further evidenced by all articles scoring at least “adequate”.

Another article investigated performance of the CKCUEST among females in the standard test position compared to a modified hands-and-knees position, finding no difference in performance between the two positions [36]. However, the preponderance of articles used the standard test position. The CKCUEST has strong, positive correlations with grip strength (*r* = 0.78–0.79) and shoulder isokinetic internal and external strength (*r* = 0.87–0.94) [37]. A moderate positive relationship was found between CKCUEST and dominant arm isometric shoulder internal rotation strength (Beta (β) = 0.38; *P* = 0.01) and trunk flexor endurance (Beta (β) = 0.26; *P* = 0.02), but a moderate negative relationship with non-dominant internal rotation range of motion (Beta (β) =  − 0.33; *P* = 0.03) [38]. CKCUEST score, normalized to body weight, was correlated with isometric strength in collegiate softball players for internal rotation (throwing/non-throwing side: *r* =  − 0.54/ − 0.57) and external rotation (throwing/non-throwing side: *r* =  − 0.58/ − 0.58; all significant at *P* < 0.01) [39]. Performance on CKCUEST was found to be moderately correlated with seated medicine ball throw (SBMT) among female non-overhead athletes (*r* = 0.69, *P* = 0.001) [21].

In a cohort of female, national-level athletes, the CKCUEST was able to identify athletes with current shoulder pain with a sensitivity of 0.86, a specificity of 0.37, positive/negative likelihood ratios of 1.36 and 0.39 (respectively) and an odds ratio of 3.53 [40]. The CKCUEST is the only upper extremity test with predictive validity for uninjured athletes: collegiate football athletes who scored less than 21 touches on CKCUEST had a sensitivity of 0.79, a specificity of 0.83, and an odds ratio of 18.75 of sustaining an in-season shoulder injury [14]. Additionally, when included in a testing battery with high school athletes, the CKCUEST demonstrated the highest specificity among components, with a sensitivity of 0.09, a specificity of 0.70, and positive/negative likelihood ratios of 0.33 and 1.28 (respectively) for identifying individuals at increased risk for injury [36]. However, when the CKCUEST was administered to elite handball players, it was unable to differentiate between players with pain, without pain, or with previous pain [36].

As the CKCUEST is a bilateral test and thus limb symmetry cannot be calculated, several articles have reported reference or normative values for healthy collegiate baseball players, stratified by positions [41], and for 28 different varsity-level collegiate sports [42]. The authors have included these for comprehensiveness only; both studies, however, are of adequate quality.

As CKCUEST validity studies varied across the range of scores, inferences can only be drawn from each specific article (Additional file 1: Appendix S1).

### Push Up

The 2-Minute Push Up Test is used as part of the US Military Service members’ physical fitness test, despite evidence that there is a lack of intra-rater (< 70%) and inter-rater (29%) agreement as to what counts as a repetition [43]. Use of a modified push up position (hands-and-knees) was found to have good reliability among healthy female recreational athletes (ICC = 0.83) [20]. Another variation of the 2-Minute Push Up Test, the Hand Release Push Up Test (HRPUT), was also found to have good reliability (ICC ranged from 0.864 to 0.881) [21]. Several articles have reported the reliability of countermovement/ballistic push-ups utilizing a force plate. Test–retest reliability was over ICC = 0.90 for most of the force plate variables (e.g., rate of force development, peak force, etc.); flight time and impulse generally had poorer reliability but varied extensively (ICC = 0.48–0.97) [44–47]. One repetition maximum (1RM) bench press performance has moderate-to-strong correlations with ballistic push-up force plate-derived variables, ranging from *r* = 0.47–0.86, in healthy male recreational athletes [46]. Short-distance swimming performance has strong correlations with an estimated 1RM push up (all *r* > 0.90) [47]. The quality of studies investigating push-ups and variations varied widely from “doubtful” to “very good” (Additional file 1: Appendix S1), with the preponderance scoring “adequate”.

### Upper Quarter Y-Balance Test

There are only three publications which report the reliability (test–retest and relative) of the Upper Quarter Y-Balance Test (UQY) [48–50] and thirteen publications examining its validity. Accompanying the original test description, the authors found that the UQY demonstrated excellent test–retest reliability for both dominant (ICC = 0.91) and non-dominant (ICC = 0.92) upper extremities in a cohort of 30 healthy college students [48]. Another study examining 29 healthy overhead athletes found that reliability ranged from ICC2,k = 0.92–0.97 [49]. However, more recent evidence reported notably lower reliability, ranging from 56.7 to 99.9% agreement across age cohort, reach arm, and reach direction in 111 healthy students, 12–17 years old [50].

Thirteen articles examined the validity of the UQY [51–63]. A study examining the relationship between UQY composite scores and the Kerlan-Jobe Orthopedic Clinic Overhead Athlete Shoulder and Elbow Score (KJOC) showed moderate, statistically significant correlations (throwing arm/non-throwing arm: *r* = 0.41, *P* = 0.01/*r* = 0.38, *P* = 0.02) [51]. The UQY demonstrated a statistically significant but weak relationship with throwing velocity (− 0.37 < *r* < 0.45) and accuracy (− 0.45 < *r* < 0.31) in Olympic handball players [52]. Another study compared the UQY (all three reach directions and composite score) to isokinetic testing (4 muscle groups, two speeds) for both limbs and found that out of 16 possible relations, the only significant correlation was between superolateral-reach direction and concentric external rotation strength at 180°/s for the non-dominant upper extremity (*r* = 0.51, *P* = 0.04) [49]. This study also found a lack of side-to-side differences for any reach direction or composite score [49]. A relationship has been noted between the anterolateral reach direction of the UQY and tennis serve speed; however, this finding is potentially spurious as only the non-dominant extremity showed a statistically significant relationship [53]. Others have reported the lack of relationship between the UQY and lower extremity power and agility testing [54], core/trunk measures [54, 55], overhead medicine ball throw [54, 55], or fastball pitching velocity [56]. In a population of professional athletes, the UQY was found to have a moderate relationships with non-dominant internal rotation range of motion (Beta (β) =  − 0.20; *P* = 0.02) [51], and dominant-side lateral trunk endurance (Beta (β) = 0.29; *P* = 0.04) [38].

When examining sex- and sport-based differences, conflicting results exist. In a cohort of Division I collegiate swimmers, male swimmers demonstrated greater reach distances by direction and composite score compared to female swimmers, all *P* < 0.05 [57]. However, no differences were found between high school male and female collegiate athletes [58]. High school wrestlers exhibit greater reach distances compared to baseball players in the medial and inferolateral direction, as well as greater composite scores, all *P* < 0.01 [59]. In a study of 418 golfers competing at the middle school, high school, collegiate and professional level, UQY performance differed by age, with golfers competing at higher levels (i.e., professional) scoring better [60]. Normative values have been reported in 665 healthy adolescents aged 10–17, stratified by age and sex [61]. Lastly, fatigue has been reported to negatively affect UQY performance [62, 63]. With the exception of two validity articles with “doubtful” scores, the preponderance of studies examining the UQY scored “adequate” or “very good”.

### Athletic Shoulder Test

The athletic shoulder test (ASH) is an emerging test designed to evaluate isometric strength of the upper body. This test involves three distinct positions: flexion, scaption, and abduction, each performed with a long lever arm. It is conducted using a force plate with the athlete in a prone position. Across all testing positions, the ASH has excellent test–retest reliability (ICCs ranging from 0.94 to 0.98) [64, 65], The ASH test also demonstrates high concurrent validity when a sphygmomanometer is utilized instead of a force plate to determine test performance (*r* = 0.76–0.82), providing a low-cost option for performing the assessment [66]. The test has also been described using a hand-held dynamometer with intersession reliability presenting as much lower than that of the original test description (ICC = 0.64–0.92). However, in a 2023 study examining performance of the ASH Test with a Kinvent hand-held push dynamometer (KINVENT, Montpellier, France), good-to-excellent reliability was shown in assessing peak force (ICCs ranged from 0.80 to 0.85) and torque (ICCs ranged from 0.84 to 0.96), as well as moderate-to-high correlation with force plate assessment of force (ICC > 0.79; *r* > 0.82) and torque (ICC ≥ 0.82; *r* > 0.76) [67]. All ASH articles scored “adequate” except one which scored “doubtful” [66], further exemplifying that this functional test is promising.

### Composite Assessments

Several articles examined a plurality of upper extremity functional tests [49, 68, 69]. In a study comparing individual functional tests to softball throw for distance in a study of 180 recreationally active subjects, each test demonstrated a statistically significant relationship of *P* < 0.01: CKCUEST: *r* = 0.33; Timed Push Up: *r* = 0.63; Modified Pull Up: *r* = 0.70; SASP (dominant/nondominant): *r* = 0.46/*r* = 0.45 [68]. The authors also used linear regression to determine which test(s) best predicted the softball throw for distance; however, the coefficient of determination was only reported for the Pull Up Test, and no *P* values indicating statistical significance were included in the publication [68].

The Shoulder Arm Return to Sport Test battery [69] consists of Ball Abduction External Rotation (BABER), Drop Catches, Ball Taps, Overhead Snatch, Pushup Claps, One-Arm Line Hops, Side Hold Rotations, and a modified version of the CKCUEST. The inter-rater reliability for all tasks ranged from ICC = 0.93–0.99, while the intra-rater reliability for all tasks ranged from ICC = 0.78–0.96. The authors also reported that the BABER (91%) and Drop Catches (93% of dominant side) showed the greatest side-to-side asymmetries, with the non-dominant extremity scoring at 91% and 93% of the dominant extremity, respectively, both *P* < 0.01 [70].

### Other Upper Extremity Functional Tests

The Seated Medicine Ball Throw is the bilateral counterpart to the SASP. One study examined the reliability of this test in 33 healthy adult overhead athletes (ICC_2,k_ = 0.98) and also compared test performance to isokinetic testing of the shoulder and elbow. The Seated Medicine Ball Throw demonstrated relationships with shoulder external rotation/internal rotation strength tested at 60°/s and 180°/s, ranging from *r* = 0.60–0.80, all *P* < 0.01 [35]. Stronger relationships were found comparing biceps and triceps strength at 60°/s and 180°/s, ranging from *r* = 0.77–0.86, all *P* < 0.01 [49]. In a study of 33 active older adults (age:72.4 ± 5.2 years), the Seated Medicine Ball Throw demonstrated excellent reliability (ICC = 0.99 for both 1.5 kg and 3 kg throws) and correlated with Explosive Push Up performance (*r* = 0.64 and 0.61 for 1.5 kg and 3 kg throws, respectively) [70]. Similar reliability was found in a study of 29 healthy female overhead athletes (ICC = 0.91) [20].

The Hand Reach Star Excursion Balance Test (HR-SEBT) is similar to the UQY; however, the individual reaches towards points in nine horizontal and rotational directions. This test demonstrates good intrarater (ICCs ranging from 0.73 to 0.90) and interrater reliability (ICCs ranging from 0.84 to 0.95) among recreationally active college students [71]. One study compared this test to team handball throwing performance (accuracy and velocity) in elite female players. Considering all reach directions, both dominant and non-dominant extremities, no significant relationship was found between hand reach star excursion balance test measures and throwing velocity. However, five of the possible 18 relationships between throwing accuracy and functional test performance (nine directions, dominant/nondominant upper extremities) were significant (*r* = 0.622–0.839; all *P* < 0.05) [72]. A separate study found moderate-to-strong correlation between the HR-SEBT and UQY (*r* = 0.32–0.76; *P* < 0.01) as well as between the HR-SEBT and CKCUEST (*r* = 0.40–0.67; *P* < 0.01). Moderate correlation was found between HR-SEBT and upper extremity maximum voluntary isometric contraction (MVIC) (*r* = 0.26–0.43; *P* < 0.05), as well as between HR-SEBT and trunk endurance tests (*r* = 0.25–0.57; *P* < 0.05) [71].

The One Arm Hop Test assesses unilateral upper extremity power in weight bearing [73]. Test–retest reliability has been reported as ICC_2,1_ = 0.81 in 13 collegiate wrestlers and ICC_2,1_ = 0.78 in 13 collegiate football players. However, no statistically significant differences were seen between dominant and non-dominant sides [73].

The Prone Medicine Ball Drop Test at 90° Abduction (PMBDT90) and Prone Medicine Ball Drop Test at 90° Shoulder Abduction/90° Elbow Flexion (PMBDT90-90) assess upper extremity endurance and control with a repeated posterior shoulder plyometric task. Test–retest reliability for PMBDT90 among healthy overhead athletes has been reported as ICC_2,1_ ranging from 0.75 to 0.82, and for PMBDT90-90 has been reported as ICC_2,1_ ranging from 0.43 to 0.81 [22]. Both of these tests also showed significant improvement in performance between the first and second testing session.

The Half Kneeling Medicine Ball Rebound Test (HKMBRT) assesses upper extremity endurance and control with a repeated anterior shoulder plyometric task. Test–retest reliability among healthy overhead athletes has been reported as ICC_2,1_ ranging from 0.85 to 0.92 [22]. This test also showed significant improvement in performance between the first and second testing session. The quality of the studies which examined these other tests ranged widely from inadequate to very good (Additional file 1: Appendix S1).

## Discussion

The primary aim of this systematic review was to evaluate the psychometric properties of upper extremity functional tests used in active individuals and athletes. Given the significant increase in attention and literature around upper extremity physical performance tests in the eight years since the 2016 review by Tarara et al. [12] and given the exclusion of multiple functional tests due to equipment needs in the 2024 review by Barbosa et al. [13] this paper is intended to provide a more expansive, updated review of current physical performance tests, including a discussion regarding their clinical relevance. The review followed PRISMA guidelines and included studies that examined the reliability and validity of these tests. Upper extremity functional tests examined included but were not limited to, the SASP, CKCUEST, UQY and the ASH test. Other functional tests were only examined in one to two studies (One Arm Hop, Hand Reach Star Excursion Balance Test, Seated Medicine Ball Throw), so confidence in their findings should be somewhat tempered. A comparative analysis between the various functional tests provides valuable insights into their psychometric properties and potential clinical applications in various settings.

The SASP demonstrated excellent reliability and validity with consistent findings across multiple studies and populations, with reliability being highest when performed from the floor. The strong relationship between the SASP and shoulder and elbow isokinetic peak torque and grip strength demonstrates its capacity to assess upper extremity strength. Meanwhile, its correlation between SASP and bench press among football players further supports its potential as a power measurement. The SASP is a valuable functional upper extremity test in sports training, rehabilitation, and injury prevention programs as it provides a reliable and valid measure of upper extremity strength and power.

The CKCUEST has exhibited good reliability and validity in a variety of different populations. The strong positive correlations between the CKCUEST and grip strength and isokinetic internal/external rotation strength indicate its utility as a strength assessment. Furthermore, the predictive validity of the CKCUEST in identifying individuals at increased risk for shoulder injury suggests its potential as a screening tool, but only in select populations. Incorporating the CKCUEST into sports or clinical settings provides insight into overall upper extremity strength, function, and potential safety with RTS.

Despite the studies which examined the UQY being well designed and executed, the test yielded mixed results regarding reliability. Westrick et al. reported excellent test–retest reliability among college students and overhead athletes [48], while Schwiertz et al. [61] indicated lower reliability rates among younger athletes. The UQY does demonstrate moderate correlations with certain measures such as the KJOC, shoulder internal rotation range of motion, and lateral trunk endurance, but weak correlation with throwing velocity and accuracy. The variability associated with the reliability and validity of the UQY requires further investigation into factors that affect performance in certain populations. Future research needs to clarify the reliability of the UQY as a potential comprehensive assessment tool for upper extremity function.

The ASH test is an emerging test with promising reliability and high concurrent validity when using a force plate or a sphygmomanometer. While early research regarding the use of a handheld dynamometer yielded a lower intersession reliability, more recent research has found the use of a handheld dynamometer to possess good reliability and concurrent validity with force plate assessment. The ASH test serves as an upper extremity functional test for assessing upper extremity isometric strength in varying positions; however, future studies should continue to examine its application in identifying injury risk and RTS readiness.

The findings of this systematic review provide insight into the psychometric properties of upper extremity functional tests. The results can inform clinicians and researchers in selecting the appropriate tests for assessing upper extremity function in active individuals and adults. However, further research is needed to continue to address gaps in literature, such as longitudinal studies evaluating the effects of these tests in specific populations and their ability to predict outcomes and injury risk. Future studies should also aim to continue to establish normative values and standardizing test protocols and interpretation of test results.

By understanding the strengths and limitations of these upper extremity functional tests, combined with the appropriateness of each test for the population of interest and sport specificity, clinicians, researchers, and practitioners can make better informed decisions regarding their selection and application of functional tests in various clinical settings. Continued research in this area will enhance the understanding of upper extremity functional testing ability to evaluate RTS decision making and contribute to the development of evidence-based prevention strategies and RTS testing.

### Clinical Relevance

The SASP test emerges as a physical performance test with excellent reliability and validity across diverse populations with consistent findings demonstrating strong relationships with shoulder and elbow isokinetic peak torques. Clinicians can consider this test for any athlete, regardless of sport or age, who needs to demonstrate upper extremity pushing power, due to its validity and reliability in assessing strength and power. With the minimal time and equipment needed to perform this test, it is an efficient way to assess the upper extremity power. This information is valuable for any open or closed kinetic chain athletes whose sport or activity requires the production of upper extremity power.

The CKCUEST emerged as a physical performance test with good reliability and validity in various populations. Strong correlations exist with grip strength and isokinetic internal and external rotation strength, highlighting its role as a comprehensive assessment tool. The CKCUEST is suitable for upper extremity athletes regardless of age and sport biomechanical demands. Its predictive validity makes the CKCUEST valuable for evaluations prior to starting Interval Sport Programs and prior to terminal evaluation. Similar to the SASP, the CKCUEST requires very minimal time and equipment to perform and provides valuable insight into the capacity and function of the upper extremity for athletes of all sports and activities.

The UQY demonstrates mixed results regarding reliability and moderate correlations with sport specific measures. There is strong correlation with this test with patient reported outcomes including KJOC Shoulder and Elbow Score and American Shoulder and Elbow Surgeons Shoulder Score but variable reliability across age cohorts prompts further investigation. The suitability of the UQY for particular sports is dependent on its reliability across the population and it should be used with caution in adolescent athletes. Given the increased time and equipment demands for this test, along with the limitations in reliability, the UQY is best served to provide insight into upper extremity function for very select sports and age groups.

The ASH demonstrates excellent test–retest reliability and high concurrent validity in assessing upper extremity isometric strength in various positions. This is a promising test, but more research needs to be completed to evaluate validity and reliability across different sport biomechanical demands and age populations. For clinicians with access to the required equipment, this test provides insight into an athlete’s ability to create force using a long lever arm in a variety of functional positions in a very short timeframe. The variety of upper extremity positions assessed with this test provides value in assessing athletes across many sports and activities.

When determining which terminal RTS physical performance tests to utilize for an athlete it is important to consider the athlete’s age, biomechanical demands and the validity and reliability of the physical performance tests. The SASP and CKCUEST emerge as two physical performance tests that, regardless of athlete age or sport biomechanical demand, demonstrate good validity and reliability with strong correlations to other upper extremity demands for a good functional assessment. These findings are consistent with a recently published Delphi study by Barber et al. [10] which utilized expert opinion to identify the CKCUEST and SASP as tests to assess RTS readiness despite sport biomechanical demands.

### Limitations

This systematic review identified multiple limitations within the current literature for upper extremity function testing. There were 60 total studies included across eight different functional tests, highlighting the overall limited amount of testing completed within the upper extremity literature. While the UQY included 15 total studies, other functional tests only included one-to-two studies (One Arm Hop, Hand Reach Star Excursion Balance Test, Seated Medicine Ball Throw). A previous systematic review published in 2016 by Tarara et al. [12] identified only 11 articles over six functional tests indicating progress with upper extremity functional test research. Additionally, the limited studies exhibited a high level of heterogeneity (demographics, competition level) and variations in testing protocols including disparities in testing instruction, procedure, and scoring. High levels of heterogeneity of testing protocols can contribute to diversity of the reliability and validity reported.

## Conclusions

Each of the various upper extremity functional tests examined in this systematic review possesses its own unique strengths and limitations. The SASP offers a reliable and valid measure of upper extremity strength and power, while the CKCUEST demonstrates strength in predictive validity of upper extremity injuries. The UQY may provide insight into upper extremity function but has significant variability across studies. The ASH test demonstrates promise in assessment of isometric upper extremity force production, but more research is necessary. The selection of upper extremity functional tests should be individualized according to the specific objectives, target population, sport specific demands and resources.

## Supplementary Information

**Additional file 1.**

## Data Availability

Summary of all included articles are in the Additional file 1: Appendix S1.

## Abbreviations

ACSM: American College of Sports Medicine
ASES: American shoulder and elbow surgeons survey
CKCUEST: Closed kinetic chain upper extremity stability test
CV: Coefficient of variation
BABER: Ball abduction external rotation
BM: Body mass
D: Dominant
DJD: Degenerative joint disease
DOR: Diagnostic odds ratio
ER: External rotation
ESI: Effect size indices
FE: Forward elevation
HHD: Hand held dynamometer
HRPUT: Hands-release push-up test
ICC: Interclass correlation coefficient
HKMBRT: Half-kneeling medicine ball rebound test
IR: Internal rotation
kg: Kilograms
Lbs: Pounds
+ LR: Positive likelihood ratio
− LR: Negative likelihood ratio
LL: Limb length
LOA: Limits of agreement
LTET: Lateral trunk endurance test
MCKCUEST: Modified closed kinetic chain upper extremity stability test
MDC: Minimal detectable change
MDD: Minimum detectable difference
MVIC: Maximum voluntary contraction
NCKCUEST: Normalized closed kinetic chain upper extremity stability test
N: Newtons
NA: Not applicable
ND: Non-dominant
PMBDT90: Prone medicine ball drop test at 90° shoulder abduction
PMBDT90-90: Prone medicine ball drop test at 90° shoulder abduction/90° elbow flexion
PT: Physical therapy
RM: Repetition maximum
ROM: Range of motion
RRI: Running-related injury
SASP: Single arm shot put
SASP-C: Single arm shot put from chair
SASP-F: Single arm shot put from floor
SD: Standard deviation
SDD: Smallest detectable difference
SEM: Standard error of measure
SIS: Subacromial impingement syndrome
SMBT: Seated medicine ball throw
TEET: Trunk extensor endurance test
TFET: Trunk flexor endurance test
UE: Upper extremity
UE-SEBT: Upper extremity star excursion balance test
ULRT: Upper limb rotation test
UQY: Upper quarter Y-balance test

## Declarations

### Disclaimer

The views expressed herein reflect the results of research conducted by the authors and do not reflect and do not necessarily reflect the views, opinions or policies of Uniformed Services University of the Health Sciences (USUHS), The Henry M. Jackson Foundation for the Advancement of Military Medicine, Inc., the Department of War (DoW) or the Departments of the Army, Navy, or Air Force. Mention of trade names, commercial products, or organizations does not imply endorsement by the U.S. Government.

### Copyright Statement

The lead author (MP) is a military Service member or employee of the U.S. Government. This work was prepared as part of this author’s official duties. Title 17, U.S.C., §105 provides that copyright protection under this title is not available for any work of the U.S. Government. Title 17, U.S.C., §101 defines a U.S. Government work as a work prepared by a military Service member or employee of the U.S. Government as part of that person’s official duties.
