# Supplementary material for: Upper Extremity Return to Sport Functional Testing: A Systematic Review
Source: Sports Med Open. 2026 Feb 23;12:13. doi: 10.1186/s40798-026-00984-4 (PMC12926263; doi:10.1186/s40798-026-00984-4)
Supplement: Supplementary file 1 — Additional file 1. [file 40798_2026_984_MOESM1_ESM.docx]

| **Reference**  **And Country** | **Study design** | **Subjects** | **Sport/Level** | **Inclusion/**  **Exclusion**  **Criteria** | **UE Functional Test(s)** | **Comparator (s)** | **Description** | **Statistical analysis** | **Results** | **Clinical Application/**  **Other** | **COSMIN Overall Grade** |
| --- | --- | --- | --- | --- | --- | --- | --- | --- | --- | --- | --- |
| **SASP Reliability** | | | | | | | | | | |  |
| Pinheiro et al^23^ | Intra and interrater reliability | 30 athletes with shoulder pain; 11 females, 19 males;  age: 23.7+4.5 years | Sports (n): weight training (7), volleyball (4), basketball (4), swimming (4), functional training (2); judo, karate, Muay Thai, rugby, capoeira, surf, badminton, handball (n=1 throughout) | Inclusion: Regular sports participation for >6 months; > 2x/week; (+) chronic shoulder pain  Exclusion: UE muscle/tendinous lesion, DJD, nerve or vascular lesions, hx UE surgery; PT within last 6 months; anti-inflammatories use in past week; rheumatologic disease. | SASP | NA | 3 trials at maximum effort per limb. Repeated at 1 week. | ICC_2,3_ with 95% CIs;  SEM;  MDC_95%_ | Intra-rater:  ICC:0.94 (0.88-0.97);  SEM (SEM%): 16.27(4.63);  MDC:45.11cm  Inter-rater:  ICC:0.97 (0.94-0.99);  SEM (SEM%): 11.64(3.37);  MDC:32.29cm | Reliable measure for individuals with shoulder pain | Adequate |
| Negrete et al^19^ | Intrarater reliability | 180 healthy recreationally active adults; 111 females, 69 males;  age:24.3 (18-45 years) | ND | Inclusion: 18-45 years old  Exclusion: recent history of UE orthopedic disorders | SASP, push up test, modified pull-up test | NA | SASP: 6lb medicine ball, performed seated with non- testing arm resting on chest, measure distance thrown (in inches)  Push-up test: repetitions completed in 15 seconds  Modified Pull-up: repetitions completed in 15 seconds | ICC_3,k_ 95% CI;  SEM;  MDC; | SASP, ND UE:  ICC:0.97 (0.97-0.98)  SEM:8 in  MDC:18 in  Push-up:  ICC:0.95 (0.90-0.97)  SEM:1 repetition  MDC:2 repetitions  Modified Pull-Up:  ICC:0.98(0.98-0.99)  SEM:1 rep  MDC:2 reps | The push-up test, modified pull-up test and SASP demonstrate excellent test-retest reliability in a healthy recreationally active population | Adequate |
| Tagliarini et al^24^ | Reliability, Validity | 44 healthy participants; 20 females, 24 males  age: 25±6.33 years | Recreationally active overhead athletes | Inclusion: 18-40 years of age, overhead athletes with a frequency of 3x/week and 1 hour/day  Exclusion: history of shoulder pain or orthopaedic surgery in the prior year, shoulder pain during shoulder abduction | SASP-F, SASP-C | SASP-F, SASP-C | Two testing sessions; 7 days between sessions  Three trials for each test | Sidak’s post hoc test  Reliability:  ICC[k,2], SEM, MDC_95_  Bland-Altman analysis (bias and 95% limits of agreement [LOA])  Pearson’s Correlation Coefficient between tests | Females demonstrate significantly greater distance with SASP-C compared to SASP-F (P<0.001)  Test-retest reliability (n=10):  SASP-F:  ICCs: 0.95-0.97  SEMs: 8.13-10.89  MDCs_95_: 22.53-30.18  SASP-C:  ICCs: 0.74-0.91  SEMs:15.76-18.94  MDCs_95_: 40.79-52.49  Presence of systematic  error found for SASP-C on D arm (P=0.011)  Validity:  SASP-F and SASP-C (total population):  Ranged from *r*=0.915 to *r*=0.990, P<0.001 | SASP is more reliable when performed from the floor, and has excellent reliability. |  |
| **SASP Validity** | | | | | | | | | | |  |
| Riemann et al^18^ | Correlational study | 24 uninjured adults; 12 females, 12 males;  age:23.7+1.9 years | Physically active >30min x 3x/week | Exclusion: self-reported trunk or UE injuries requiring surgery or medical attention | SASP | Isokinetic pushing force | 1 session;  SASP: 3 trials per limb with 0.97 and 2.27 kg ball; concentric isokinetic testing at 0.24, 0.43, 0.61 m/s | Pearson correlation coefficients;  Bland-Altman analysis (bias and 95% limits of agreement [LOA]) | SASP distances and isokinetic peak forces for both limbs, all speeds: r= 0.755-0.868, all P<0.01  Bland-Altman analysis: greater limb symmetry indices for the SASP than the isokinetic ratios (P<0.1); biases: 0.094 to -0.159  95% LOA: ±0.241 to ±0.340 for 0.91 kg ball; ±0.202 to ±0.221 for 2.27kg balls | Significant moderate to strong relationships were revealed between the SASP and isokinetic peak forces at all speeds for both medicine balls and limbs | Doubtful |
| Watson et al^25^ | Concurrent validity | 30 active and uninjured adults; 15 females, 15 males;  age: 25.0+3.4 years | Physically active >30min x 3x/week x 3 months | Exclusion: pre- existing cervical spine or UE pain, injury, or surgery within 12 months | SASP, isokinetic shoulder flexion and elbow extension strength | NA | 1 session; after standard warm-up, SASP: 3 trials per limb with 2 kg ball; concentric isokinetic testing at 60° and 180°/s | Pearson correlation coefficients;  Bland-Altman analysis: bias and 95% LOA | Strong relationships between SASP ranges and isokinetic peak torques at each velocity for both shoulder and elbow (*r*≥.804, P<.01);  LSI: significant bias for the shoulder (60°/s, P<.01)  LOA: extremely wide intervals (32.5%–52.1%) | The SASP test is reflective of shoulder flexion and elbow extension function; however, there was large variability between SASP LSI and isokinetic shoulder and elbow strength LSI. | Adequate |
| Mayhew et al^27^ | Concurrent validity | 64 healthy female athletes;  age: 19.6±1.2 years | Volleyball (N=7), basketball (N=21), soccer (N=18), tennis (N=6), softball (N=12); college varsity level | Not specified | SASP | Bench press power | One testing session, 7 anaerobic power tests, order randomized  SASP: 3 trials, 4.5kg indoor shot  Bench Press Power: 3 maximal effort trials with absolute mass of 20kg through 0.19m | Pearson correlation coefficients | Seated shot put and bench press power:  *r=*.38, P<.05 | There is low correlation between seated shot put and bench press power, rendering it difficult to infer upper extremity power from this test | Doubtful |
| Mayhew et al^28^ | Concurrent Validity | 40 healthy male athletes; age: 20.4 + 1.4 years | NCAA DII football players | Inclusion Criteria:  ND  Exclusion Criteria:  ND | Shot Put Test | 1-Repetition Maximum (RM) Bench Press | 1 testing session;  1 RM bench press: 3 attempts to reach 1 RM  Absolute power: absolute load of 61kg for each subject  Relative power: 60% of subjects 1RM  Shot put test: 4.5kg ball; score= average 2 farthest throws (m) | Concurrent Validity:  Pearson correlation coefficients | Shot put test and absolute power *r=0.51*  Shot put test and relative power *r=0.66* | The shot put test was found to be significantly related to absolute and relative power via the 1 RM bench press test in healthy NCAA DII football players | Doubtful |
| Pinheiro et al^26^ | Construct Validity | 19 participants; 10 females, 9 males  age: 26.8±5.4 years | Recreational athletes | Inclusion: physically active, aged 18-45 years, nonspecific shoulder pain ≥3/10 on NRPS for > 3 months, positive test for two orthopaedic tests (Neer, Hawkins-Kennedy, Jobe, painful arc)  Exclusion: specific musculoskeletal injury; ligament laxity in the UE, use of medication, surgery, or PT in the last 6 months; neurovascular injury of the UE; diagnosis of fibromyalgia | SASP | UE strength, UE ROM | One testing session  Three trials for each measurement | Spearman correlation coefficient | No correlation between SASP and shoulder ROM except for horizontal adduction (r=0.465, P=0.045)  SASP and UE strength:  Flexors: r=0.549  Extensors: r=0.575  Abductors: r=0.567  Adductors: r=0.616  Internal rotators: r=0.672  External rotators: r=0.470  Grip: r=0.833  (all P<0.05) | SASPT correlates highly with grip strength, and moderately with other UE strength measures. SASP has little to no relationship with shoulder ROM | Adequate |
| **CKCUEST Reliability** | | | | | | | | | | |  |
| Callaway et al^30^ | Reliability- internal consistency | 34 healthy male participants | ND | Inclusion: Injury free at the time of testing | CKCUEST | Modified Closed Kinetic Chain Upper Extremity Stability test | 4 sessions; 1 session per week over 4 weeks; Hand positions test: 36 inches, shoulder breadth, shoulder breadth to 36 inch touch, 50% body height; measure= number of touches | ICC_3k_ with 95%CI;  SEM;  MDC; | 36 inches:  ICC: 0.90(0.73-0.96)  SEM: 1.73 touches  MDC: 4.76 touches  Shoulder breadth:  ICC: 0.84(0.71-0.92)  SEM: 2.85 touches  MDC: 7.78 touches  Shoulder breadth to 36”:  ICC: 0.89(0.81-0.94)  SEM: 2.06 touches  MDC: 5.72 touches  50% height:  ICC: 0.93(0.87-0.97)  SEM: 1.49 touches  MDC: 4.14 touches | All hand positions demonstrated excellent internal reliability with the CKCUEST in healthy male participants | Adequate |
| Goldbeck et al^34^ | Reliability | 24 males;  mean age: 20.3 years | Healthy college students | Inclusion: college male students | Closed Kinetic Chain Upper Extremity Stability Test | NA | 2 sessions; 7 days apart.  1 submax warmup  3 maximal tests; 45 second rest between tests  2 max tests were averaged | Single measure ICC’s  Paired samples correlation coefficient  Coefficient of stability (r²) | ICC: 0.922  Correlation coefficient: 0.927  Coefficient of stability (r):0.859 | The results indicated the CKCUEST is reliable and can be used as an upper extremity functional test | Adequate |
| Tucci et al^29^ | Reliability | 108 healthy sedentary subjects, age 20-65 years; 20M/20 F healthy sedentary adults; 20M/20 F healthy recreational athletes;  13M/15F sedentary adults with impingement | Sedentary: performed less than 30 minutes of daily physical activity; physically active: performed 1 or more physical activity at least three times/week in last 3 months including 1 specific UE activity | Inclusion, SIS group: shoulder pain > 3 months, (+) shoulder impingement tests, painful arc, <65 years old  Inclusion, healthy subjects: sedentary or UE specific recreational athletes, no history of shoulder pain/surgery  Exclusion:  history of shoulder or traumatic injury to trunk, elbow or hand; rheumatoid, neurologic, or degenerative disease | CKCUEST | NA | 2 sessions; 7 days apart; scoring number of touches | Reliability: Intersession:  ICC_2,3_,  Intrasession: ICC_2,1_  SEM;  MDC | Inter-session:  Sedentary male: ICC:0.96  Sedentary female: ICC:0.92  Active male: ICC: 0.89  Active female: ICC: 0.85  Male SIS: ICC: 0.91  Female SIS: ICC: 0.93  Intra-session:  Sedentary male: ICC:0.96; SEM: 1.45; MDC: 2.05  Sedentary female: ICC:0.97; SEM: 2.43; MDC: 3.34  Active male: ICC: 0.95; SEM: 2.0; MDC: 2.82  Active female: ICC: 0.95; SEM: 2.76; MDC:3.91  Male SIS: ICC: 0.97; SEM: 1.95; MDC:2.76;  Female SIS: ICC: 0.92; SEM: 1.89; MDC:2.67 | All scores were greater in active compared to sedentary and individuals with SIS  CKCUEST demonstrates excellent inter and intrasession reliability in recreational athletes, sedentary individuals with and without SIS | Very good |
| Declève et al^31^ | Intra-session and test-retest reliability and concurrent validity | 73 healthy adolescents; basketball players (39); volleyball players (34);  age: 14.7 + 1.4 years | Good general health and played a minimum of sport 3 hours per week | Exclusion: history of orthopedic surgery of the upper quadrant, spine or reports of pain in these areas within a 6- month period prior to the study | Modified Closed Kinetic Chain Upper Extremity Stability Test | Self Assessment Corner | 2 sessions; 7 days apart; Modified CKCUEST: males and females from pushup position; hand position: inter-acromial distance; measure: number of touches | Intra-session Reliability: ICC_2,1_ with 95% CI  Test-retest reliability ICC_3,1_  MDC  SEM  Pearson product moment correlation | Intra-session reliability:  ICC_2,1_: Day 2= 0.86 (0.80- 0.90)  ICC_2,1_: Day 2- 0.89 (0.81-0.93)  Test-retest reliability: ICC_3,1_:0.93 (0.63- 0.97)  MDC 3.04 touches;  SEM: 1.1 touches;  Correlation: *r*: 0.3-0.4 | Good to excellent relative reliability and absolute reliability values for the MCKCUEST  Performances on the MCKCUEST were weakly associated with shoulder rotation strength | Adequate |
| Tarara et al^33^ | Test-retest and interrater reliability | 19 healthy physically active college-aged students; 11 females; 8 males;  age: females 19.8+1.0 years; males 20.3+1.2 years | Maintained physically active cardiorespiratory endurance for 3 or more days per week and/or 2 or more days per week of progress resistive exercise program | Inclusion: currently active in club sport season or have participated in past year  Exclusion: sustained an orthopedic injury within past 6 months, pre-existing cardiovascular, pulmonary or metabolic disease | CKCUEST | NA | 2 sessions, separated by 4-7 days; measure number of touches | Test-retest and interrater reliability: ICC_3,1_ with 95% CI  SEM; | Test-retest:  ICC= 0.73 (0.41-0.89);  Interrater reliability:  Expert rater: ICC:0.73(0.41-0.89); SEM: 8.00 touches;  Novice Rater:  ICC:0.78(0.47-0.92); SEM: 6.92 touches;  Novice rater 2:  ICC: 0.77(0.50-0.91); SEM: 7.12 touches | The CKCUEST demonstrates good to excellent test retest and interrater reliability in a healthy active college population | Very good |
| Sciascia et al^32^ | Test-retest reliability | 36 subjects; 18 asymptomatic (10 females, 8 males); 18 symptomatic (9 females, 9 males)  age: asymptomatic: 29+ 7 years; symptomatic 30+ 8 years | NA | Asymptomatic inclusion: >90 on ASES, no pain > 2/10, no limitation in ROM, no point tenderness, no (+) findings for tissue derangement/ other conditions  Symptomatic inclusion: pain > 3/10; ASES score < 89  Exclusion criteria: pain >8/10 ; current condition medically disqualifying the individual or currently participating in a postsurgical rehabilitation program, demonstrated signs of cervical radiculopathy, or had shoulder and/or neck surgery in the past 24 months. | 1 RM estimate of scapular plane elevation strength test;  CKCUEST;  Isometric UE strength testing | NA | 2 sessions; 7-10 days apart  CKCUEST measured number of touches;  Isometric strength test: in the scapular plane (90° and 30° horizontal abduction), 3 maximum effort reps;  force output in kg  1 RM scaption strength:  10 max effort reps; maintain cadence of 47 beats/minute, measured weight utilized | ICC_2,1_, SEM and MDC at 95% CI;  Independent t-tests utilized for between group comparisons | Asymptomatic group:  Isometric test D: ICC:0.98; SEM: 1kg; MDC: 1kg;  Scaption ND:  ICC: 0.98; SEM: 1kg; MDC: 1kg;  CKCUEST:  ICC: 0.85; SEM: 2 touches; MDC: 4 touches;  1-RM D:  ICC: 0.96; SEM: 1 kg; MDC: 2 kg;  1-RM ND:  ICC: 0.94; SEM: 1 kg; MDC: 2kg  Symptomatic Group:  Isometric test D: ICC:0.97; SEM: 1kg; MDC: 2kg;  Scaption ND:  ICC: 0.95; SEM: 1kg; MDC: 2kg;  CKCUEST:  ICC: 0.86; SEM: 2 touches; MDC: 4 touches;   1. RM D:   ICC: 0.93; SEM: 1 kg; MDC: 3 kg;  1-RM ND:  ICC: 0.93; SEM: 1 kg; MDC: 3kg  Between group comparisons:  Isometric Task D:  P=0.89; Isometric Task ND: P=0.99;  CKCUEST: P=0.06; 1-RM estimate D: P=0.36; 1-RM estimate ND: P=0.17 | These tests demonstrated excellent test/retest reliability in symptomatic and asymptomatic males and females; These tests are not effective in discriminating between symptomatic and asymptomatic individuals | Very good |
| Degot et al^17^ | Reliability | 27 healthy athletes; 27 males;  age: 22.5±3.2 years | Rugby (N=11), judo (N=5), soccer (N=3), fitness (N=2), basketball (N=2), climbing (N=1), volleyball (N=1), yoga (N=1), running (N=1); weekly training 8.6±5.8 hours | Inclusion: 18-30 years of age and asymptomatic upper extremities  Exclusion: upper extremity injury within 6 months or history of upper extremity surgery | MCKCUEST | NA | Two sessions, one week apart;  Tape lines placed at a distance of half of the participant’s arm span;  Three trials of MCKCUEST performed, followed by a fourth 1-minute trial in which the examiner counted touches in each 15-secon period | ICC[3,k], SEM, MDC_95_ for inter- and intrasession reliability for sets | Intrasession reliability of sets 1-3; all p<0.05:  ICC: 0.86-0.95  SEM: 0.55-0.89 touches  MDC_95_: 1.52-2.45 touches  Intersession reliability of sets 1-3; all p<0.05:  ICC for sets 1-3:0.68- 0.89  ICC for means of sets 1-3: 0.83-0.92  SEM for sets 1-3: 0.78- 1.29 touches  SEM for means of sets 1-3: 0.68-0.90 touches  MDC_95_ for sets 1-3: 2.13-3.57 touches  MDC_95_ for means of sets 1-3: 1.87-2.48 touches  Intersession reliability of sets 4-7; all p<0.05:  ICC for sets 4-7: 0.48- 0.80  ICC for means of sets 4-7: 0.59-0.87  SEM for sets 4-7: 1.42- 2.03 touches  SEM for means of sets 4-7: 1.22-1.66 touches  MDC_95_ for sets 4-7: 3.93-5.61 touches  MDC_95_ for means of sets 4-7: 3.32-4.1 touches | mCKCUEST has good reliability not only in three 15-second trials, but also in a fourth 1-minute trial to assess muscular endurance | Adequate |
| **CKCUEST Validity** | | | | | | | | | | |  |
| Lee et al^37^ | Reliability and validity | 40 healthy Korean adults; 20 females; 20 males;  age: 28.96 + 3.15 years | ND | Inclusion: no pain or disability in hand grip; no pain, instability or limited range of motion in shoulder function | CKCUEST | NA | 2 session; 3 days between sessions;  Grip strength: 3 max strength trials;  Isokinetic strength IR and ER testing: concentric at 60° and 180°/s | ICC for test-retest reliability with 95% CI;  Pearson correlation coefficients | ICC: 0.97 (0.93-0.99)  Grip strength and CKCUEST:  Right= *r:* 0.79 P=<0.01  Left=*r*: 0.78 P<0.01  Isokinetic IR/ER 60°/s and CKCUEST:  R IR= *r*:0.90 P=<0.01  R ER=*r:*0.90 P=<0.01  L IR= *r*: 0.93 P=<0.01  L ER= *r*:0.91 P=<0.01  Isokinetic IR/ER 180°/s and CKCUEST:  R IR= *r*:0.94 P=<0.01  R ER=*r:0*.82 P=<0.01  L IR= *r*: 0.90 P=<0.01  L ER= *r*:0.87 P=<0.01 | The reliability of the CKCUEST was excellent  The correlations between the CKCUEST and maximum grip strength and the peak torque of internal/external shoulder rotation were high indicating its validity | Adequate |
| Pontillo et al^14^ | Validity | 26 collegiate varsity football players; 26 males;  mean age: 19.6 years | Football; collegiate varsity | Inclusion: football athletes who currently played at the varsity level at a Division I university  Exclusion: current upper extremity or cervical spine injury | CKCUEST  Isometric strength of shoulder forward elevation and ER at 90deg ABD  Fatigue testing battery | NA | All testing performed in 1 session;  CKCUEST: 2 trials;  Isometric strength of shoulder elevation and ER at 90 deg ABD via handheld dynamometer strapped against immovable object, 2 trials each position;  Fatigue testing via cable press, all 1 trial, repetitions to failure using a percentage of BW as resistance;  Standing cable press: 30% BW  Scaption: 5% BW  Prone Y: 3% BW | Multiple logistic regression assessed if variables predicted whether an athlete would sustain a shoulder injury the season following testing  Receiver operator characteristic (ROC) curve analysis used to determine cutoff scores for significant predictors  Sensitivity, specificity, + LR, -LR, and odds ratio calculated based on the cutoff scores (95% CI). | FE strength (P=0.05), prone-Y to fatigue (P=0.05), and the CKCUEST (P=0.05) independent predictors of right shoulder injury; CKCUEST of left shoulder injury (P=0.03)  ROC analysis: AUC=0.75 for FE strength (P=0.05; 95% CI= 0.58-0.92) and 0.70 for prone-Y to fatigue (P=0.11; 95% CI=0.46-0.94) ;  AUC=0.86 for CKCUEST (P<0.01; 95% CI=0.72-1.0);    To predict injury, CKCUEST score <21.0 touches:  Sensitivity: 0.79 (0.57-0.91)  Specificity: 0.83 (0.44-0.97)  +LR: 4.74 (0.78-28.78)  -LR: 0.25 (0.10-0.65)  DOR: 18.75 (1.68-209.55) | CKCUEST, included within a pre-season battery of tests, can help in identifying individuals at increased risk of UE injury | Adequate |
| Powell et al^35^ | Validity | 13 healthy athletes; 8 females, 5 males; age: 19.7±5.15 years | Canoe/kayak slalom; national level | Inclusion: member of the British Canoe/Kayak Slalom program  Exclusion: any musculoskeletal injury at time of testing or symptoms during testing | CKCUEST  MCKCUEST | NA | All testing performed in 1 session;  CKCUEST, MCKCUEST: 3 trials each with order of start position randomized;  Start position: CKCUEST: 36 inches  MCKCUEST: arm length;  All measured in number of touches | Pearson’s correlation coefficient  Independent t-test for differences between sexes for MCKCUEST  Paired samples t-test for differences between in scores for narrow and wide hand positions | CKCUEST scores and arm length:  CKCUEST: *r*=0.81, P<0.01  MCKCUEST: *r*=0.10, P=0.75  NCKCUEST: *r*=0.654, P=0.02    MCKCUEST and sex:  Mean difference: 0.4  95% CI=-2.2-2.9; P=0.77  Difference in CKCUEST scores for start positions:  Mean difference: 3.2  95% CI=1.8-4.5, P=0.00  Narrow start position averaged more touches | As arm length has a strong relationship with CKCUEST score, using MCKCUEST may better describe an athlete’s abilities | Inadequate |
| Terry et al^74^ | Predictive Validity | 133 healthy athletes;  age: 18.6±1.31 years | Multiple sports (exact numbers not described); high school level | NA | CKCUEST | NA | One testing session in which CKCUEST was performed as part of a seven-task battery;    A score of 2 was provided if the athlete achieved 20+ touches, 1 if the athlete achieved <20 touches, and 0 if any pain present | Sensitivity, specificity, LRs, odds ratios for ability to predict injury | CKCUEST to predict injury:  Sensitivity: 0.09  Specificity: 0.70  +LR: 0.33  -LR: 1.28  Odds Ratio: 0.25 (0.09-0.66) | CKCUEST possesses second highest specificity among all components of testing battery, indicating that it may be useful in identifying individuals at increased risk of injury | Very good |
| Gaudet et al^40^ | Validity | 34 healthy female athletes;  age: 21.7±5.2 years | Synchronized swimming and team handball; national level | Inclusion: Every active athlete attending their respective team selection trials, regardless of shoulder pain status or history;    Exclusion: current injury preventing them from training or completing the CKCUEST at time of testing | CKCUEST | NA | 1 testing session.    Athletes reported whether they were: (1) playing with no pain, (2) playing with pain, or (3) not playing due to pain, then completed CKCUEST over two AccuGait force platforms | k-means clustering; 2x2 contingency tables;  Sensitivity, Specificity, +LR, -LR, DOR for CKCUEST touches, and CKCUEST time to peak GRF to identify injured athletes | Clustering using CKCUEST touches:  Sensitivity: 0.86  Specificity: 0.37  +LR: 1.36  -LR: 0.39  DOR: 3.53 | CKCUEST has high sensitivity and low specificity in identifying injured athletes | Inadequate |
| Schilling et al^39^ | Concurrent validity Normative values | 74 healthy athletes; 24 females/ 50 males  age: females 19.2±1.0 years; males 19.3±1.0 years | Baseball (n=50) and softball (n=24) Collegiate level | Inclusion: baseball or softball collegiate athletes; over 18 years of age; no current shoulder injury or medical conditions that precluded them from participation in sports | CKCUEST | Isometric shoulder ER and IR strength in 90-0 position | One testing session;  Isometric strength testing:  ER and IR in prone 90-0 position using HHD  CKCUEST:  Three trials performed, recording total number of touches | Pearson product-moment correlation coefficient: relationship between strength and CKCUEST  Independent t-tests: compare shoulder strength and CKCUEST scores between baseball and softball players | No correlation between shoulder IR/ER strength and CKCUEST score or power in baseball or softball players;  CKCUEST power and IR strength in softball players:  Throwing side:  *r* = -0.54, P< 0.01  Non-throwing side: *r*=-0.57, P< 0.01  CKCUEST power and ER strength in softball players:  Throwing side:  *r*=-0.58, P< 0.01  Non-throwing side:  *r=*-0.58, P< 0.01 | CKCUEST score is not correlated to shoulder strength in baseball or softball players  CKCUEST power has moderate correlation to ER and IR strength in softball players | Adequate |
| Torabi et al^36^ | Construct Validity | 106 healthy athletes; 49 females/57 males  Age: females 22±4.9 years; males 22±5.4 years | Handball; elite level | Inclusion: healthy handball players participating in both offensive and defensive portions of matches and training  Exclusion: recovering from injury, excluded from activity in the last six weeks, current/previous shoulder pain secondary to traumatic injury or surgery | CKCUEST | NA | One testing session  Three recorded trials; 15 female subjects performed from knees if unable to perform in standard position | Pearson’s correlation coefficient: comparing subject height and CKCUEST raw score, power score, and touches  One-way ANOVA: female subjects performing CKCUEST versus modified  Two-way ANOVA: compare subjects with pain, previous pain, or no pain; compare males and females | Correlation found between height and CKCUEST power score in male and female groups  Males: *r* = 0.66, P<0.01  Females: *r* = 0.43, P=0.01  No significant difference between females performance standard or modified CKCUEST  No significant differences between pain groups and raw score, touch score, and power score  Significant differences between male and female subjects (P<0.01):  Raw score: F=125, η^2^=0.59  Touch score: F=107, η^2^=0.71  Power score: F=82.6, η^2^=0.48 | CKCUEST performance appears to be influenced by height and sex.  Female performance on CKCUEST and modified CKCUEST is comparable.  CKCUEST cannot differentiate among handball players who are playing with pain, previous pain, and no pain | Very good |
| **CKCUEST- Normative** | | | | | | | | | | |  |
| Roush et al^41^ | Normative values | 77 healthy male athletes;  age: 19.03±1.2 years | Baseball; collegiate level | Exclusion: outside of age range (18-22y), surgery on either UE in the last 12 months, not fully cleared by their team physician, pain or fatigue in either upper extremity from recent activity | CKCUEST | NA | One testing session;  Three trials of CKCUEST | Means (SDs); one-way ANOVA | Means by position:  Pitcher: 30.30 (SD 4.82) touches  Catcher: 30.41 (SD 3.52) touches  Infielder: 30.78 (SD 4.02) touches  Outfielder: 30.30 (SD 4.00) touches  All Players: 30.41 (SD 3.87) touches  ANOVA:  Non-significant | Normative values can be used in return to sport algorithm with no significant difference between positions | Adequate |
| Pontillo et al^42^ | Normative values | 476 healthy athletes; 206 females; 270 males;  mean age: 18 years | All male and female varsity sports; collegiate level | Exclusion: current or previous cervical spine or upper extremity injury, or current upper extremity or cervical pain | CKCUEST | NA | One testing session;  Two trials of CKCUEST | Means (SDs) | Mean scores males/females = 26.0 (4.1) touches / 21.8 (3.9) touches  Normative values for all male sports:  Lowest=golf  Highest= football  Normative values for all female sports:  Lowest= squash  Highest= gymnastics | Normative values can be used as part of return to sport algorithm; recommendation to return to plyometrics when within 2 SD of norms, and return to sport when within 1 SD of norms | Adequate |
| **Push Up Reliability** | | | | | | | | | | |  |
| Parry et al^45^ | Test-retest Reliability | 18 elite male boxers;  age: 23+ 3 years | Elite boxers; flyweights (n=2), bantamweights(n=2), lightweights (n=6), welterweight (n=1) middleweights (n=2), light heavyweight(n=1), heavy weights (n=2), super heavy weights (n=1) | NA | Countermovement push-up | NA | 2 sessions; 7 days apart;  Power measurements: flight time (ms), peak force (N), mean force (N), rate force development (RFD; N^.^s), impulse (N^.^s), vertical stiffness (kN/m) | ICCs with 95% CI;  within subject CV%;  SEM;  SDD;  Mann-Whitney U test for side- to-side differences | Test-retest  Flight time:  ICC: 0.76(0.48-0.65); CV%: 6.9; SEM:0.1; SDD: 0.2;  Peak force:  ICC: 0.92 (0.82-0.90); CV%: 4; SEM: 53.6; SDD: 148.5;  Mean force:  ICC: 0.97(0.89-0.94); CV%:3; SEM: 14.9; SDD:41.2;  RFD:  ICC: 0.79 (0.53-0.92); CV%: 8; SEM: 239.1; SDD: 662.6;  Impulse:  ICC: 0.94(0.84-0.97); CV%: 8; SEM: 8.2; SDD: 22.6;  Vertical Stiffness:  ICC: 0.56(0.15-0.26) CV%: 16.5; SEM: 0.6; SDD: 1.7 | Force platform-derived countermovement push-ups demonstrate moderate to high reliability in elite-level boxers  No significant difference between right and left limbs for all parameters | Doubtful |
| Fielitz et al^43^ | Reliability | 15 healthy US Military Academy members; 3 females; 12 males;  age:21+1 year | Military training | NA | 2-Minute Push Up Test | NA | 4 review sessions; 30 seconds between each 2- minute push-up test; minimum of 7 days between review sessions | Inter-rater and Intra-rater reliability were evaluated using Pearson product-moment correlation, kappa, modified kappa (M-kappa) and ICC 3,1 | Inter-rater reliability coefficients: 0.10- 0.97  Intra-rater coefficients: 0.48- 0.99  Intra-rater agreement for individual push-up repetitions: 41.8%-84.8% | Raters failed to assess the same push-up repetition with the same score as well as failed to agree when viewed between raters | Very good |
| Parry et al^44^ | Test-retest reliability | 10 healthy male college athletes;  age:24+3 years | Minimum of 1 year of Olympic lifting training | NA | Countermovement push up | NA | 2 testing sessions; 7 days apart; power parameters: flight time (seconds), peak force (N), mean force (N), rate of force development (N/second), impulse (newton seconds) | ICC;  Coefficient of variation with 95% CI;  SEM;  SDD | Flight time:  ICC: 0.96; CV95%: 6.9: SEM: 0.0; SDD: 0.0;  Peak Force:  ICC: 0.96; CV95%: 3.9; SEM: 31.1 SDD: 86.3;  Mean Force:  ICC: .96; CV95%: 1.7; SEM: 1.7; SDD: 50.3  Rate Force Development:  ICC: .87; CV95%: 14.1; SEM: 105.1; SDD: 291.3  Impulse:  ICC: .98; CV95%: 5.5; SEM: 7.1; SDD: 19.7 | Force platform-derived kinetic parameters of countermovement push-ups demonstrate moderate to high reliability for measures of power in college-level athletes | Adequate |
| Fanning et al^75^ | Reliability, concurrent validity | 39 healthy athletes; 39 males;  age: 18-40 years  Reliability study: 21 subjects from original group of 39 | Competitive collision/contact sports; local, regional, or national level | Inclusion: participation in competitive collision/contact sports  Exclusion: upper limb pathology within 6 months; upper limb surgery in the previous 12 months | Countermovement pushup, press jump, box drop landing | Isokinetic strength of shoulder ER and IR at 90° elevation | 2 testing sessions (second session 2-9 days later to assess reliability);    Countermovement pushup, box drop landing, and press jump: 3 trials each;  Isokinetic testing of IR/ER at 90° abduction: 2 sets of 5 repetitions | Reliability: ICC (95% CI);  Concurrent validity:  Pearson correlation coefficient | Reliability:  ICCs 0.80-0.97 for all-time series data for three UE performance tests except for landing impulse of countermovement pushup and box drops (ICCs 0.67-0.79)  Concurrent validity:  No significant relationship between peak IR/ER torque and any variables of performance tests | The upper extremity plyometric performance tests of countermovement pushup, box drop landing, and press jump are reliable, but appear to have no relationship with peak shoulder IR/ER torque | Adequate |
| Wang et al^46^ | Reliability;  concurrent validity | 60 healthy male subjects;  age: 24.5±4.3 years | Recreationally active | NA | Ballistic pushup | One repetition maximum bench press test | Three testing sessions;  Each session included 1RM testing and two trials of ballistic pushup on force plate | Reliability:  ICC (95% CI)  Validity:  Pearson correlation coefficient | Reliability:  Ballistic pushup (ICC):  Peak force: 0.97 (0.95-0.98);  Mean force: 0.99 (0.98-0.99);  Peak RFD: 0.84 (0.76-0.91)  Mean RFD: 0.87 (0.78-0.92)  Net impulse: 0.96 (0.93-0.97)  Peak velocity: 0.86 (0.78-0.92)  Flight time: 0.75 (0.61-0.84)  Peak power: 0.94 (0.90-0.96)  Mean power: 0.93 (0.89-0.96)  Validity (all P<0.01):  Peak force: *r*=0.85;  Mean force: *r*=0.86;  Peak RFD: *r*=0.65;  Mean RFD: *r*=0.64;  Net impulse: *r*=0.82;  Peak velocity: *r*=0.47;  Flight time: *r*=0.24;  Peak power: *r*=0.75;  Mean power: *r*=0.74; | The ballistic pushup has good-to-excellent reliability and has moderate-to-strong correlation with 1RM bench press performance | Adequate |
| **Push Up Validity** | | | | | | | | | | |  |
| Amara et al^47^ | Concurrent validity | 33 healthy male athletes;  age:16.5±0.59 years | Swimming at a national level | NA | Estimated 1 RM pushup | Swimming performance (25m and 50m front crawl, 25m and 50m front crawl with arms only), kinematic variables of 50m front crawl | One testing session;  Pushup test performed on force plate with four different loads, three repetitions each; 1RM estimated based on force-velocity equation;  25m, 50m front crawl, and 25m, 50m front crawl with arms only all performed once | Pearson correlation coefficient | Swimming performance (seconds) and 1RM pushup:  25m front crawl:  *r*=-0.94  50m front crawl:  *r*=-0.97  25m front crawl arms only: *r*=-0.94  50m front crawl arms only: *r*=-0.96  Swimming kinematics and 1RM pushup:  Velocity: *r*=0.96  Stroke length: *r*=-0.93  Stroke rate: *r*=0.96  Stroke index: *r*=0.56 | An estimated 1RM pushup has a strong correlation to swimming performance in short distances | Doubtful |
| **Upper Quarter Y Reliability** | | | | | | | | | | |  |
| Westrick et al^48^ | Reliability | 30 healthy subjects; 6 females; 24 males;  age: females 18.8 + 0.8 years; males 19.5 + 1.2 | College students | Exclusion: UE disability via SPADI/DASH | UQY | NA | 2 sessions; minimum of 2 weeks following the initial assessment. | ICCs and within-subject coefficient of variation within 95% CI | Test re-test reliability values were similar for both dominant (ICC=0.91) and non-dominant (ICC=0.92) | The UQYBT is a reliable test that can be used to assess unilateral UE function in a closed chain fashion | Very good |
| Schwiertz et al^50^ | Reliability | 111 healthy subjects; 52 females, 59 males;  age: 12-17 years | Students in grades 6-11 | Inclusion: 12-17 years old;  Exclusion: had musculoskeletal or neurological disorder; other medical condition that could affect that ability; could not perform the retest | UQY | NA | 2 sessions separated by 1 week. | Relative reliability was assessed using ICC_3,1_ and 95% confidence intervals | Irrespective of age cohort, reach arm, and reach direction, the ICC3,1 value ranged from “moderate-to-good” to “excellent  Left: 85.4-99.9% (medial), 72.8-92.7% (inferolateral), and 58.8-66.3% (superolateral)  Right: 84.3-98.3% (medial), 72.3-91.0% (inferolateral), and 56.7-63.0% (superolateral) | The detected values imply that the UQY is a reliable field test that can be used to detect changes of upper quarter mobility and stability in healthy adolescents aged 12-17 years. | Adequate |
| **Upper Quarter Y Validity** | | | | | | | | | | |  |
| Stapleton et al^54^ | Validity | 38 healthy athletes; 15 females; 23 males  age: females 19.9±1.3 years; males 20.0±1.4 years | College baseball/ softball | Exclusion: Injury at the time of the study; somatosensory disorder; any low back pain; medication that affect balance; pregnancy | UQY | Athletic performance: vertical jump; pro-agility; medicine ball throw | 1 session;  3 trials, best score taken, composite score taken and limb length normalized | Pearson correlation coefficient P<0.05 | Baseball players: no significant  correlations between composite FMS, YBT-LQ, UQY, and any of the athletic performance tests: composite scores did not significantly predict total performance for either sex/sports |  | Very good |
| Singla et al^55^ | Validity | 48 healthy males  age: adolescent age 16.4±1.0 years; adult age 20.9±1.7 years | Cricket | Exclusion: Any injury, fatigue, pain or history of surgery in past 6 months | UQY | Body strength overhead medicine ball throw | 1 session;  UQY: 3 trials, average score taken, composite score taken and limb length normalized;  Back strength: digital back muscle dynamometer;  Med ball throw: 3kg ball thrown overhead as far as possible | Pearson correlation coefficient P<0.05 | No correlation between UQY and back strength;  Adolescents: 0.05(.81) D and -0.01 (.78) ND;  Adults: 0.33 (0.14)D and 0.41 (0.06)ND  Nonsignificant correlations between UQY and power. | This study did not find a correlation between UQY with muscle strength or power in adolescent and adult cricketers. | Adequate |
| Salo et al^63^ | Validity | 24 healthy participants; 7 females; 17 males  age: 25.8± 2.7 years | College age weightlifters | Inclusion: performed UE resistance weight training an average of 3 days a week  Exclusion: red flag in medical chart; prior surgical history to UE; current pain | UQY | UQY post fatigue protocol | 1 session: 2x 3 trials, average and composite score taken, normalized to limb length;  Experimental group performed fatigue protocol between sets of trials | Two-way factorial ANOVA | Experimental group demonstrated a significant reduction in UQY directional and composite scores post fatigue protocol and compared to the non-fatigue protocol group (all P<0.05). | The performance of an upper body fatigue protocol significantly reduces YBT-UQ scores in recreational weightlifters. | Adequate |
| Palmer et al^53^ | Validity | 42 healthy males  age: 23.9±5.8 years | Competitive tennis players | Exclusion: current medical treatment for injury; National Tennis Rating Program 4.5 or higher | UQY | UQY and Serve speed | 1 session;  3 trials, average score taken, composite score taken and limb length normalized | Pearson correlation coefficient P<0.05 | Significant positive correlation with the UQY of the ND arm for the anterolateral reach direction.  *r*=0.33, P=0.02 | Only non-dominant arm anterolateral reach exhibited significant correlation to serve speed. Sample did not show significant correlations for other UQY directions in the UE exhibiting lack of usefulness of the test for predicting serve speed. | Very good |
| Bauer et al^52^ | Validity | 56 healthy participants; 14 females; 24 males;  age: females 13 years (training experience 5.9± 1.3 years); males 14 years (training experience (6.5 ± 2.5 years) | Youth select team handball players | Exclusions: any injury in prior 2 weeks; visual, vestibular, and proprioceptive disorders | UQY | UQY and throwing proficiency (speed/ accuracy) | UQY- 3 trials, best score taken, composite score normalized to limb length  Throwing- standard net and ball for each sex; 3 tasks (velocity, accuracy, corner targets) x 3 trials each | Paired t-tests;  p-value of < 0.05;  Pearson’s correlation coefficient; 0 ≤ r ≤ 0.69 indicate small, 0.70 ≤ r ≤ 0.89 medium, and r ≥ 0.90 large sizes of correlation | Small correlations between UQY performance and throwing velocity/accuracy (13-year-old females:−0.01≤*r*≤ −0.37 / 0.01≤*r*≤0.31; 14-year-old males: 0.10≤*r*≤0.45/ -0.01≤*r*≤.-0.51; 15-year-old males: 0.06≤*r*≤0.34 / 0.01≤*r*≤−0.45 | There was only a minimal difference in performance of the UQY between limbs. Weak relationship between throwing performance and UQY. | Adequate |
| Bullock et al^56^ | Validity | 30 healthy males;  age: 20.4+ 1.5 years | Collegiate baseball pitchers | Exclusion: current injury; pain during testing; restrictions with sports | UQY | UQY  trunk rotations and velocity | 1 session:  UQY- 3 reaches with rests and max score, composite score;  Velocity- dynamic warmup followed by “usual pitching session;” only fastballs considered | 2-tail Pearson’s correlations. P<0.05 | No statistically significant correlations between UQY and pitching velocity.  D medial: 0.2 (0.3); inferolateral: 0.2 (0.19); superolateral: 0.3 (0.1); composite: 0.3 (0.1);  ND medial: 0.3 (0.2); inferolateral 0.1 (0.8); superolateral 0.3 (0.2); composite: 0.2 (0.2) | UQY is not directly related to fastball velocity. | Adequate |
| Butler et al^57^ | Validity | 97 healthy participants; 54 females; 43 males  age: females 19.1±0.7 years; males 19.3±1.2 years | NCAA Division I collegiate swimmers | Exclusion: Pain, under medical care for injury Injury in past 6 months | UQY | Sex differences | 1 session;  3 reaches;  Max score to composite score | Independent sample t-test at P<0.05 | Male swimmers exhibited greater reach in medial (males: 100± 8.8% LL; females: 92.5 ± 8.1% LL; ESI = 0.89), inferolateral (males: 89.8 ± 10.8 LL; females: 85.6 ± 10.3 LL; ESI = 0.40), and composite score reaches (males: 88.3 ± 8.9 LL; females: 83.4 ± 8.3 LL; ESI: 0.57) | Male swimmers exhibited greater dynamic closed chain upper quarter function on the UQY for independent reach directions as well as for the composite score. | Very good |
| Butler et al^58^ | Validity | 65 healthy participants; 17 females; 48 males  age: females 15.2±1.1 years; males 15.8±1.2 years | High School baseball and softball | Exclusion: pain; pain during testing; known injury | UQY | Throwing vs. non-throwing side | 1 session;  3 reaches;  Max score to composite score | Independent sample t-test at P<0.05 | No differences were found for any of the reach directions or the composite score between the throwing and non-throwing sides, P=0.42‐0.91, ESI: 0.01‐0.08 | UQY performance does not differ between limbs in overhead throwing high school athletes. | Adequate |
| Myers et al^59^ | Validity | 48 healthy males;  age: wrestlers 16.12±1.24 years; baseball players 15.79±1.25 years | High school wrestlers and baseball | Exclusion: no upper extremity pain in the past 6 month | UQY | Closed chain sport vs open chain sport | 1 session;  3 reaches;  Max score to composite score | Independent sample t-test at P<0.05 | Wrestlers exhibited significantly greater reach distances than baseball players in the medial direction: [L: 10.54 ± 10.20; R: 9.95 ± 10.20 (P< .01)], inferolateral direction [L: 11.31 ± 12.00; R: 8.70 ± 11.00 (P< 0.01)], and for the overall composite score [L: 7.00; R: 7.12 (P< 0.01)]. | This study suggests that wrestlers perform better on the UQY than baseball players. | Doubtful |
| Bauer et al^62^ | Validity | 24 healthy male participants;  age: 14.8±0.7 years | Youth handball | NA | UQY | Fatigued vs. not fatigued | UQY 1 session;  3 reaches;  Max score to composite score;  Fatigue protocol;  Retest | Paired t-test  P<.005 | Post fatigue protocol: decreases in superolateral reach (throwing arm reach −5%, P=0.01; throwing arm reach: −10%, P < 0.01) and composite score (throwing arm reach: −2%, P=0.03; non-throwing arm reach: −4%, P<0.01).  Post fatigue protocol: no decrement for medial or the inferolateral reach | Fatigue was found to be an impairing factor for throwing performance and UQY performance. | Doubtful |
| Chasse et al^51^ | Validity | 36 healthy male participants;  Previously injured, surgical (n=9): 21.1±1.7 years; previously Injured, non-surgical (n=6): 20.0±0.9; uninjured (n=21): 20.2±1.3 years | College baseball pitchers | Exclusion: pain with testing or with baseball; current injury | UQY | UQY;  Trunk rotation  KJOC | 1 session:  Administer KJOC prior to session;  Seated trunk rotation with rod;  UQY: 3 reaches;  Max score to composite score | Pearson’s Correlation Coefficients: P<0.05  One-way ANOVAs P<0.05 | Positive relationship with UQY composite scores and the KJOC composite scores.  throwing arm: *r*=0.41, P=0.01; non-throwing arm: =.38, P=0.02  No significant differences were observed between throwing and non-throwing arms across groups in measures of trunk rotation (*r*= 0.71, P= 0.38) or UQY (*r*=0.73, P=0.91) | A positive moderate association was found between upper quarter dynamic stability as measured by the UQY and the KJOC. | Adequate |
| Krysak et al^60^ | Validity | 418 healthy male participants; 53 middle school; 129 high school; 207 college age; 29 professional  mean age: middle school: 12.22 ± 2.43 years; high school: 15.95 ± 1.27 years; 19.55 ± 1.62 years; professional: 31.75 ± 11.80 years | Golfers | Excluded: any injury in past 3 months, history of concussion or vestibular problems. Any pain during testing | UQY | UQY (and LQY and FMS) differences across levels | 1 session;  3 reaches;  Max score to composite score | Dunn's post-hoc was implemented to identify specific group-to-group differences  p<0.05 | Professional golfers’ medial reaches were significantly greater than college (P<0.01), high school (P<0.01) and middle school (P<0.01).  Professional golfers’ inferolateral and superolateral reaches were statistically greater when compared to college (p=0.04) and high school (P<0.01).  Composite scores were significantly higher in professional golfers compared to college (P<0.01), high school (P<0.01) and middle school (P<0.01)  College golfers’ medial reaches were significantly higher compared to high school (P=0.0003) and middle school (P=0.0325) reaches. | The results of the current study indicate that there are differences in golfers’ movement patterns in relationship to their skill level. The collective findings highlight that skill level should be taken into consideration when interpreting test results. | Adequate |
| Schwiertz et al^50^ | Validity | 665 healthy subjects; 325 females; 340 males  age: 56 10–11-year-olds; 175 12-13-year-olds; 218 14–15-year-olds; 216 16–17-year-olds. | Adolescents | Inclusion: Age 10-17    Exclusion: musculoskeletal or neurological disorder; medical condition that restricted | UQY | Age norms | 1 session;    3 trials, best score taken, composite score taken and limb length normalized | Mean values ± standard deviations;  Multivariate analyses of variance (MANOVA) (p > 0.05) | UQY reach performance by % arm length:  Medial: 94.9%-100.7%  Inferolateral: 83.1%-98.4%  Superolateral: 60.9%-76.8%  Composite: 80.1%-92.6% | The obtained age-and sex-specific normative values for the UQY can be used by teachers, coaches, and therapists to classify the level of shoulder mobility and stability among 10–17 year-old children and adolescents. | N/A; compared normative values for the age groups |
| **Athletic Shoulder Test (ASH)** | | | | | | | | | | |  |
| Ashworth et al^64^ | Test- retest reliability | 18 healthy males;  age: 22.4+ 4.6 years) | Elite rugby players | Exclusion: Sustained an acute injury to the neck or shoulder girdle (<72 hours prior to testing), symptoms after performing the test that lasted longer than 20 minutes or worsening pain attributed to the test, or unable to complete the test secondary to inadequate range of motion | Athletic Shoulder Test (ASH test) | NA | 2 testing sessions; measured as peak force | ICC with 95% CI | ICC: D Arm  I position=0.97 (.93-.99)  Y position=0.96 (0.92-0.98)  T position= 0.98 (0.95-0.99);  ICC: ND Arm  I position= 0.95(0.89-0.98)  Y position= 0.94 (0.87-0.97)  T position= 0.96 (0.92-0.98) | Excellent test-retest reliability across all 3 testing positions in a healthy made population | Adequate |
| Morrison et al^66^ | Concurrent validity | 20 healthy male athletes;  age: 25.4±3.3 years | Amateur rugby players | Exclusion: acute injury to the neck or shoulder girdle which prevented participants from playing or training (<3 days prior to testing); if participants complained of pain during the test or if limited range of motion prevented them from adopting any of the test positions | Sphygmomanometer strength via ASH test | Force plates | 1 testing session; force plate measured in newtons; sphygmomanometer measured in millimeters of mercury | Pearson’s coefficient of correlation with 95% CI | I: *r*= 0.770 (0.64-0.90)  Y: *r*= 0.817 (0.66-0.93)  T: *r*= 0.764 (0.64-0.9) | High concurrent validity found between Sphygmomanometer and hand held dynamometer for the ASH test | Doubtful |
| Tooth et al^65^ | Reliability and validity | 20 healthy male subjects;  age: 22.1+ 2.1 years | Tennis, volleyball, badminton, handball, CrossFit; training 8.3 + 5.5 hours per week for 3 years | Inclusion: NA  Exclusion:  Asymmetry of lower limb length; scoliosis or dorsal hyper-kyphosis; shoulder surgical history; shoulder pain or shoulder injury | Modified Athletic Shoulder Test | Athletic Shoulder Test | 2 sessions, 7 days apart; Modified athletic shoulder test: 3 maximal trails after 3 submaximal trials in each position (I, Y and T) with a handheld dynamometer, measured kg ASH test: measure force in N | Concurrent Validity:  ICCs and  Bland-Altman analysis to determine bias and 95% LOA  Intersession Reliability:  ICC, SEM and MDC | Concurrent Validity:  ICC: I position= 0.95 (0.88-0.98) P=0.86  ICC: Y Position= 0.93 (0.58-0.97) P=0.61  ICC: T position= 0.84 (0.69-0.94) P=0.11  Intersession Reliability:  ICC: I position= 0.92 (0.82-0.92) P= 0.19  ICC: Y Position= 0.64 (0.23-0.85) P=0.01  ICC: T position= 0.83 (0.60-0.93) P=0.06 | Good to excellent concurrent validity between ASH and modified ASH test  I position is most reliable and T position being least reliable  Poor intersession reliability for ASH and modified ASH test in healthy male subjects | Adequate |
| Olds et al^67^ | Reliability and validity | 27 healthy participants; 17 females, 10 males  age: 37±12 years | Healthy population | Inclusion: 18-55 years of age  Exclusion: presence of shoulder pain or any musculoskeletal or neurological condition of the UE | Athletic Shoulder Test (ASH Test) via HHD | Athletic Shoulder Test (ASH Test) via force plates | One testing session  Three trials for each measurement | Reliability:  ICCs[2,3], SEM, MDC_95_  Concurrent Validity:  Pearson correlation coefficient, ICCs | Reliability:  Force:  ICCs: 0.80-0.95  SEMs: 0.66-1.4  MDCs_95_: 1.71-3.87  Torque:  ICCs: 0.84-0.96  SEMs: 3.42-8.06  MDCs_95_: 9.49-22.35  Normalized torque:  ICCs: 0.64- 0.93  SEMs:0.04-0.13  MDCs_95_: 0.12-0.35  Concurrent validity:  Force:  *r*≥0.82  ICCs: 0.79-0.93  Measuring torque:  *r*≥0.76  ICCs: 0.82-0.94  Measuring normalized torque:  *r* ≥0.61  ICCs: 0.7-0.89  No significant differences across trials on analyses of variance | The ASH Test is reliable when Kinvent HHD is used to assess UE force, torque, and normalized torque, and possesses concurrent validity with force plates.  Lack of significant differences across trials suggests that clinicians can perform test for one trial instead of averaging three trials | Adequate |
| **Other Tests** | | | | | | | | | | |  |
| Harris et al^70^ | Reliability and validity | 33 community dwelling older adults; 13 females; 20 males;  age: 72.4+5.2 years | Community dwelling older adults performing resistance training 2x per week | Inclusion: part of a senior resistance training class  Exclusion:  ND | Seated Medicine Ball Throw | Explosive push up | 2 testing sessions; 3 trials of throws for 2 weights (1.5 and 3.0 kg) measured distance thrown cm | Product-moment correlation;  ICC; | Reliability of 1.5 kg throw:   *r*=0.967  ICC coefficient *r*=0.994  SEM: 19.1 cm  Reliability of 3.0 kg throw:  *r*=0.9875  ICC coefficient *r*= 0.989  SEM 14.8 cm  Validation of seated medicine ball throw  for 1.5 kg ball: =0.641  3.0 kg ball: *r*=0.614 | For older adults the Seated Medicine Ball test is a highly reliable test of UE power with moderate validity to maximal force of explosive push up. | Adequate |
| Falsone et al^73^ | Test re-test reliability | 26 healthy males; 13 college wrestlers; 13 college football players;  age: wresters 20.3±1.6 years; football players 20.0±1.7 years | College wrestlers and football players | Exclusion: no upper extremity injury currently limiting athletic participation and no history of upper extremity trauma or injury having limited athletic activity for more than 2 consecutive days during a 6-week period prior to testing. | One-arm hop test | NA | 2 sessions, 1-2 days apart;  measured time in seconds | Test-retest reliability: ICC _2,1_  D versus ND side: 2-way ANOVA test | Test-retest reliability: ICC_2,1_: Wrestler= 0.81  ICC_2,1_: Football=0.78;  ANOVA: subject group and UE dominance did not interact significantly to affect one-arm hop test scores (F=0.63; *df*=1,24).  One-arm hop test scores did not differ significantly between dominant and non-dominant UE (F=1.92; *df*=1,24) | Good to excellent test re-test reliability;  Unable to conclude a difference in power between dominant and non-dominant extremities | Doubtful |
| Eriksrud et al^72^ | Concurrent validity | 11 healthy females;  age: 21.7 + 1.8 years | Elite International handball players | Exclusion: Musculoskeletal or neurologic dysfunction or injury in the past six months, inability to participate in normal handball and throwing activities and pain or discomfort reported during testing | Hand reach Star Excursion Balance Test | Throwing velocity and accuracy | 1 session; hand reach excursion test (cm)  Throwing protocol: A 3-step run-up throw from 8m was used with a one minute rest period between throws. Subjects kept throwing until 5 valid throws inside the target was obtained | Pearson correlation | Dominant:  R45^o^= *r:* 0.12 P=0.71  L45^o^ = *r*: 0.48 P=0.12  L135^o^ = *r*: 0.72 P=0.01  R135^o^ = *r*: 0.698 P=0.01  RROT= *r*:0.12 P=0.78  LROT= *r*:0.07 P=0.83  CS= *r*:0.62; P=0.04  CS flex= *r*: 0.33 P=0.31  CS EXT= *r*:0.75 P=<0.01  Non-Dominant:  R45^o^= *r:*0.36 P=0.27  L45^o^= *r*: <0.01 P=0.97  L135^o^= *r*: 0.48P=0.13  R135^o^= *r*: 0.83 P=<0.01  RROT= *r*:0.07 P=0.82  LROT= *r*:0.22 P=0.50  CS= *r*:0.58 P=0.59  CSflex= *r*: 0.23 P=0.48  csEXT= *r*:0.65 P=0.02 | No significant correlations between throwing velocity and individual hand reach star excursion balance reaches or composite scores were observed in healthy female handball players; | Doubtful |
| Olds et al^61^ | Reliability | 40 healthy participants; 20 females; 20 males  age: male 25.3±6.86 years; female 23.8±4.78 years | College students | Inclusion: 18-55 years of age  Exclusion: injury to the upper extremity, back or neck within 12 months, history of shoulder or spine surgery, upper extremity fracture, ongoing neck pain, neurological symptoms in any extremity, low back, hip or knee pain | Ball Abduction External Rotation (BABER), drop catches, ball taps, overhead snatch, pushup claps, one-arm line hops, side hold rotations, CKCUEST, | NA | Test-retest design of three tests, one week apart;  Inter-rater reliability examined between three raters on Day 2, while intra-rater of an expert rater was examined across three time points. | ICC of each rater across three days, SEM and MDC with 90% CI | Inter-rater reliability for all tasks:  ICC=0.93-0.99  SEM=1-2  MDC_90_=1-3  Intra-rater reliability for all tasks:  ICCs=0.78-0.96  SEM=1-4  MDC_90_=0-10  BABER (91% of D side) and Drop Catches (93% of D side) significantly decreased on ND side (P<0.01) | Six of eight tasks of SARTS battery demonstrate good reliability and appear to have good clinical utility in guiding return to sport decisions  Asymmetry between D/ND arms may be expected with BABER and drop catch tests | Very good |
| Borms et al^49^ | Validity;  reliability | 29 healthy athletes; 15 females; 14 males  age: age=21.6±2.5 years | Volleyball (N=16), basketball (N=8), badminton (N=3), handball (N=1), volleyball (N=1), tennis (N=1); varying levels of sport, participating in at least 3 hours of overhead sports per week | Inclusion: age 18-50 and participating in overhead sports at least 3 hours/week  Exclusion: history of orthopaedic surgery or pain of the upper quarter, lower quarter, or spine in the six months prior to the study | SMBT, UQY | Isokinetic strength for shoulder ER/IR, and elbow flexion/extension | 1 testing session;  SMBT: 2kg medicine ball, four trials;  UQY: 3 test trials executed for each limb in each direction;  Isokinetic testing: shoulder ER/IR conc-conc, shoulder ER conc-ecc, max elbow flex/ext conc-conc, elbow flexion conc-ecc | Reliability:  ICCs [2,k]  Validity:  Pearson’s coefficient of correlation | SMBT:  Reliability:  ICC [2,k]=0.98  SEM=10.82cm  MDC_95_=29.98cm  Validity:  Shoulder ER/IR at 60°/s and SMBT:  Ranged from r=0.75 to r=0.80; all P<0.01  Shoulder ER/IR at 180°/s and SMBT:  Ranged from r=0.60 to 0.74; all P<0.01  Shoulder ER/IR (60° and 180°/s) and SMBT:  Ranged from r=0.60 to r=0.80; all P<0.01  Elbow biceps/triceps at 60°/s and SMBT:  Ranged from r=0.77 to 0.86; all P<0.01  Elbow biceps/triceps at 180°/s and SMBT:  Ranged from r=0.80 to 0.85; all P<0.01  Elbow biceps/triceps (60° and 180°/s) and SMBT:  Ranged from r=0.77 to r=0.86; all P<0.01  UQY:  Reliability:  ICCs [2,k] = 0.924-0.967  Concentric ER at 180°/s and superolateral reach: *r*=0.51 P=0.04 | SMBT is a reliable option to assess UQ strength in clinical setting due to moderate-to-strong correlations with shoulder and elbow strength  UQY should not be used in isolation to assess shoulder or elbow strength | Adequate |
| Negrete et al^60^ | Concurrent validity  Predictive validity | 180 healthy participants; 111 females; 69 males  age: 24.3±5.33 years | Recreationally active | Exclusion: currently involved in competitive throwing related activities, history of upper extremity injury or surgery | CKCUEST, Timed Pushup, Timed Modified Pull-Up, SASP, softball throw for distance | CKCUEST/Timed Pushup/ Timed Modified Pull-Up/ SASP/ softball throw for distance | Multicenter study  1 testing session  SASP: 2.72kg ball, three trials each arm  Timed Pushup: males performed in hands-and-toes position, females performed in hands-and-knees position; three trials  Modified Pull-Up Test: subject positioned supine, men performed with support at heels, women performed with support just below knee; three trials    CKCUEST: three trials  Softball Throw for Distance: standard league softball, one-step approach to throwing; three maximum effort throws | Two-tailed Pearson correlation  Stepwise multiple regression analysis | Concurrent Validity:  Functional test and softball throw distance:  Ranged from r=  0.33 to r=70; all P<0.01  CKCUEST: *r*=0.33 P<0.01  Timed Pushup: *r*=0.63 P<0.01  Modified Pull-up: *r*=0.70 P<0.01  D SASP:  *r*=0.46 P<0.01  ND SASP:  *r*=0.45 P<0.01  Predictive validity for softball throw distance:  Ranged from r^2^=0.487 to r^2^=0.675  Modified Pull-Up Test: r^2^=0.487  Modified Pull-Up Test, weight: r^2^=0.645  Modified Pull-Up, weight, and height: r^2^=0.662  Modified Pull-up, weight, height, and age: r^2^=0.675 | Modified Pull-Up Test has a moderate-to-high correlation with softball throw distance and may play a role in predicting throwing performance | Very good |
| Kardor et al^20^ | Reliability | 29 healthy females  age: 26.6 + 5.29 years | Recreational athletes;  Volleyball (n=10)  Basketball (n=9)  Badminton (n=10) | Inclusion: female athletes age 18-35 years, active in overhead sports, no UE injury that limited activity for more than two consecutive days in the previous six months, no systemic or neuromuscular disease  Exclusion: history of upper extremity or spinal surgery, use of hypnotic and sedative medication | Modified Pushup Test, CKCUEST, SMBT, SASPT | NA | Two testing sessions with three-day rest between sessions  Modified pushup:  Hands-and-knees position; as many repetitions as possible in three sets with 45-second rest between sets  CKCUEST:  Three trials  SMBT:  Three trials with one-minute rest between trials  SASPT:  Three trials with one-minute rest between trias | ICC[2,1] across the two sessions, SEM, MDC | Test-retest reliability:  Modified Pushup:  ICC= 0.83, SEM=1.69, MDC_95_=4.68  CKCUEST:  ICC= 0.80, SEM=1.72, MDC_95_=4.75  SMBT:  ICC= 0.91, SEM=52.12cm, MDC_95_=144.04cm  SASPT (D arm):  ICC= 0.65, SEM=21.36cm, MDC_95_=59.03cm  SASPT (ND arm):  ICC= 0.83, SEM=13.61cm, MDC_95_=37.62cm | Modified pushup, CKCUEST, SMBT, and SASPT (ND) all have excellent test-retest intra-rater reliability in the female overhead athlete. SASPT (D) has moderate test-retest reliability in the female overhead athlete. | Adequate |
| Riemann and Davies^21^ | Reliability | 40 healthy participants; 20 females; 20 males  age: females: 23.9±2.0 years; males: 25.2±3.2 years | Participants all had history of non-overhead sport participation; Weightlifting (n=8), Flag Football/Football (n=9), Running (n=8), Basketball (n=6), Soccer (n=4), Cheerleading (n=1), Golf (n=2), Gymnastics (n=1), Wrestling (n=1) | Inclusion: 18-35 years of age, meeting ACSM criteria for being physically active, participated in a non-overhead sport for a minimum of one year  Exclusion: previous cervical spine or UE injury within a year prior to study, deficient ROM needed to perform tests, unable to complete tests and prescribed | CKCUEST, SMBT, HRPUT | NA | Two testing sessions with three-to-seven days rest between sessions  Order of tests randomized  CKCUEST:  Three trials  SMBT:  Three trials  HRPUT:  One trial | Dependent t-tests  ICC[2,1] across the two testing sessions, SEM, MDD_90_, CV%  Pearson correlation coefficient between three tests | Test-retest reliability:  Significant improvement between sessions on CKCUEST for males (P=0.003) and females (P=<0.001)    CKCUEST:  ICCs: 0.823-0.954  SEMs: 1.0 to 1.4  MDDs_90_: 2.3-3.3  CV%: 5.0-6.0  SMBT:  ICCs: 0.84-0.90  SEMs: 0.19-0.29  MDDs_90_: 0.45-0.67  CV%: 4.5-5.1  HRPUT:  ICCs ranged from 0.86-0.88  SEMs: 2.7-3.3  MDDs_90_: 6.4-7.6  CV%: 8.8-12.5  No significant associations between tests except CKCUEST and SMBT in females (*r*=0.691, P=0.001) | The CKCUEST demonstrates performance improvement in the second testing session, suggesting possible learning effect.  The CKCUEST, SMBT, and HRPUT all demonstrate good-excellent reliability.  Performance on CKCUEST and SMBT is moderately correlated among females. | Very Good |
| Riemann et al^22^ | Reliability | 40 healthy participants; 20 females; 20 males  age: females: 23.2±1.7 years; males: 24.5±2.6 years | Participants with history of overhead sport participation; Softball (n=15), Baseball (n=7), Swimming (n=2), Tennis (n=3), Volleyball (n=10), Football (quarterback) (n=3) | Inclusion: 18-35 years of age, meeting ACSM criteria for being physically active, participated in an overhead sport for a minimum of one year  Exclusion: previous cervical spine or UE injury within a year prior to study, deficient ROM needed to perform tests, unable to complete tests and prescribed | PMBDT90, PMBDT90-90, HKMBRT, SASPT | NA | Two testing sessions with three-to-seven days rest between sessions  Order of tests randomized  PMBDT90:  One trial  PMBDT90-90:  One trial  HKMBRT:  One trial  SASPT:  Three trials | Dependent t-tests  ICC[2,1] across the two testing sessions, SEM, MDD_90_, CV% | Test-retest reliability:  All tests demonstrated significant improvement between sessions except for SASPT (P≤0.03)  PMBDT90:  ICC: 0.75-0.82  SEM: 5.6-6.5  MDD_90_: 13.1-15.1  CV: 7.3-10.7%  PMBDT90-90:  ICC: 0.43-0.81  SEM: 5.2-7.8  MDD_90:_: 12.2-18.2  CV: 10.3-17.6%  HKMBRT:  ICC: 0.85-0.92  SEM: 3.8-5.7  MDD_90_: 8.9-13.9  CV: 5.5-9.4%  SASPT:  ICC: 0.85-0.96  SEM: 0.13-0.17  MDD_90_: 0.30-0.40  CV: 3.0-5.3% | The PMBDT90, PMBDT90-90, HKMBRT, and SASPT all possess acceptable reliability and can be used for serial assessments of UE function. | SASPT: Very good  HKMBRT: adequate  PMBDT90: Doubtful  PMBDT90-90 Inadequate |
| Yang et al^71^ | Reliability; validity | 65 healthy participants; 36 females, 29 males  age: females: 22.08±2.32 males: 21.24±2.03 | Recreationally active college students | Inclusion: 18-35 years of age, no injury history within three months with pain during tests, normal cognitive function  Exclusion: cardiac or mental health related conditions, disobedient behavior during the test | UE-SEBT | UQY, MVIC of UE, CKCUEST, TFET, TEET, LTET | Two testing sessions with 48 hours of rest between sessions  UE-SEBT, UQY, MVIC:  Three trials  TFET, TEET, LTET:  One trial | Reliability: ICC[3.1], SEM, MDC_95_  Validity:  Two-tailed Spearman test | Intrarater reliability:  ICC ranged from 0.729 to 0.899.  Interrater reliability:  ICC ranged from 0.837 to 0.946  Validity:  UE-SEBT and UQY:  *r*=0.315-0.755, P<0.01  UE-SEBT at CKCUEST: *r*=0.4-0.67, P<0.01  UE-SEBT and UE MVIC:  *r*=0.26-0.43, P<0.05  UE-SEBT and TFET:  *r*= 0.25-0.33, P<0.05  UE-SEBT and LTET:  *r*=0.26-0.57, P<0.05  UE-SEBT and TEET:  *r*=0.27-0.44, P<0.05 | The UE-SEBT has good reliability, and demonstrates moderate to strong correlation with UQY and CKCUEST, as well as carrying correlation with trunk endurance tests and UE MVIC. It can be used to serially assess UE stability | Very good |
| Kara et al^38^ | Validity | 121 healthy participants; 71 females, 50 males  age: 20.97±3.07 years | Professional athletes;  Basketball (n=25), Volleyball (n=44), Handball (n=52) | Inclusion: healthy professional overhead athlete, 18+ years of age, engage in athletic training 3+ days s week, and understand written/oral information  Exclusion: previous orthopaedic surgery, injury in the last six months, presence of neurological disease, presence of pain during testing | CKCUEST, UQY, ULRT | Glenohumeral IR, ER, HADD ROM, Thoracic rotation ROM, isometric strength of glenohumeral ER and IR musculature, trunk muscle endurance (flexors, extensors, lateral flexors) | One testing session.  Three trials for each measurement | Regression using SMDM-estimator | IR ROM of the nondominant side was associated with the CKCUEST, the YBT-UQ, and the ULRT. IR muscle strength of the dominant side was associated with the CKCUEST and the ULRT.  Trunk flexor and lateral endurance of the dominant side were associated with the CKCUEST and the YBT-UQ, respectively.  Adjusted R^2^ ranged from 0.10-0.23; all analyses had P<0.05 | The CKCUEST and UQY appear to assess different aspects of physical performance, so combined use is recommended. | Doubtful |

**Abbreviations:**

ACSM= American College of Sports Medicine, ASES= American Shoulder and Elbow Surgeons Survey, CKCUEST= closed kinetic chain upper extremity stability test, CV= coefficient of variation, BABER= Ball Abduction External Rotation, BM-Body mass, D=dominant, DJD=degenerative joint disease, DOR = diagnostic odds ratio, ER= external rotation, ESI= Effect size indices, FE = forward elevation, HHD= Hand Held Dynamometer, HRPUT= Hands-Release Push-Up Test, ICC= interclass correlation coefficient, HKMBRT= Half-Kneeling Medicine Ball Rebound Test, IR= internal rotation, kG= kilograms, Lbs= pounds, +LR = positive likelihood ratio, -LR = negative likelihood ratio, LL= limb length; LOA=limits of agreement, LTET= Lateral Trunk Endurance Test, MCKCUEST = modified closed kinetic chain upper extremity stability test, MDC= minimal detectable change, MDD= minimum detectable difference, MVIC= maximum voluntary contraction, NCKCUEST = normalized closed kinetic chain upper extremity stability test, N= newtons, NA=not applicable, ND=non-dominant, PMBDT90= Prone Medicine Ball Drop Test at 90° Shoulder Abduction, PMBDT90-90= Prone Medicine Ball Drop Test at 90° Shoulder Abduction/90° Elbow Flexion, PT=physical therapy, RM= Repetition Maximum, ROM= Range of Motion, RRI=running-related injury, SASP= single arm shot put, SASP-C= single arm shot put from chair, SASP-F= single arm shot put from floor, SD= standard deviation, SDD= smallest detectable difference, SEM= standard error of measure, SIS= subacromial impingement syndrome, SMBT=Seated Medicine Ball Throw, TEET= Trunk Extensor Endurance Test, TFET= Trunk Flexor Endurance Test, UE= upper extremity, UE-SEBT= Upper Extremity Star Excursion Balance Test, ULRT= Upper Limb Rotation Test, UQY= upper quarter Y-balance test,
